# α-Ketoglutarate promotes trophectoderm induction and maturation from naive human embryonic stem cells

**DOI:** 10.1038/s41556-025-01658-1

**Published:** 2025-04-23

**Authors:** Karlien Van Nerum, Anne Wenzel, Lidia Argemi-Muntadas, Eleni Kafkia, Antar Drews, Ida Sophie Brun, Viktoria Lavro, Annina Roelofsen, Nikolaos Stamidis, Sandra Bages Arnal, Cheng Zhao, Simone di Sanzo, Moritz Völker-Albert, Sophie Petropoulos, Thomas Moritz, Jan Jakub Żylicz

**Affiliations:** 1 Novo Nordisk Foundation Center for Stem Cell Medicine – reNEW, Department of Biomedical Sciences, Faculty of Health and Medical Science, University of Copenhagen, Copenhagen, Denmark; 2 Novo Nordisk Foundation Center for Basic Metabolic Research, University of Copenhagen, Copenhagen, Denmark; 3 Department of Clinical Sciences, Intervention and Technology, Karolinska Institutet, Stockholm, Sweden; 4 MoleQlar Analytics GmbH, Munich, Germany; 5 Centre de Recherche du Centre Hospitalier de l’Université de Montréal, Axe Immunopathologie, Montreal, Quebec Canada; 6 Département de Médecine, Université de Montréal, Montreal, Quebec Canada; 7 Department of Gynecology and Reproductive Medicine, Karolinska University Hospital, Stockholm, Sweden

**Keywords:** Embryonic stem cells, Embryology

## Abstract

Development and lineage choice are driven by interconnected transcriptional, epigenetic and metabolic changes. Specific metabolites, such as α-ketoglutarate (αKG), function as signalling molecules affecting the activity of chromatin-modifying enzymes. However, how metabolism coordinates cell-state changes, especially in human pre-implantation development, remains unclear. Here we uncover that inducing naive human embryonic stem cells towards the trophectoderm lineage results in considerable metabolic rewiring, characterized by αKG accumulation. Elevated αKG levels potentiate the capacity of naive embryonic stem cells to specify towards the trophectoderm lineage. Moreover, increased αKG levels promote blastoid polarization and trophectoderm maturation. αKG supplementation does not affect global histone methylation levels; rather, it decreases acetyl-CoA availability, reduces histone acetyltransferase activity and weakens the pluripotency network. We propose that metabolism functions as a positive feedback loop aiding in trophectoderm fate induction and maturation, highlighting that global metabolic rewiring can promote specificity in cell fate decisions through intricate regulation of signalling and chromatin.

## Abstract (web summary)

Van Nerum et al. show that dimethyl α-ketoglutarate enhances the production and functional maturation of human trophectoderm-like cells from embryonic stem cells by regulating histone acetyltransferase activity and the pluripotency network.

## Main

The first week of human development is coordinated by a complex interplay of signalling pathways and chromatin regulatory processes. This elaborate coordination is particularly evident after compaction, when cells of the early pre-implantation embryo undergo the first fate decision to either establish pluripotency within the inner cell mass (ICM) or specify towards the trophectoderm (TE) lineage. This lineage choice is predominantly driven by cell polarity and the Hippo pathway, a regulatory axis that is broadly conserved across mammals, including humans^1–3^. The fundamental signalling asymmetry between inner and outer blastomeres allows the induction of the gene regulatory networks (GRNs) driving either naive pluripotency or the opposing TE programme, respectively^4,5^. The TE plays a vital role during human blastocyst development and subsequent implantation as its polar fraction attaches and invades the endometrium^6,7^. Human implantation constitutes an important developmental bottleneck, with a substantial percentage of blastocysts failing to establish pregnancy^8^. The competence of the human blastocyst to implant probably depends on the robust maturation of the polar TE, which begins to express a specific GRN marked by for example, *NR2F2* and *GCM1* (refs. ^9,10^).

While using human pre-implantation embryos in research poses substantial hurdles, human naive embryonic stem (nES) cells enable the study of key aspects of early human development. These cells stabilize the naive pluripotency GRN while maintaining the potential to acquire the identity of extra-embryonic lineages, including the TE and later trophoblast fate^11–19^. Indeed, nES cells can establish human induced trophoblast stem (hiTS) cells^20^. Moreover, nES cells readily self-organize into TE- and epiblast (Epi)-containing blastoids, which are in vitro models resembling the human pre-implantation blastocyst^21–24^. Similar to the human embryo, Hippo signalling is also crucial in this context^21^. Along with direct transcriptional regulation through signalling, additional epigenetic pathways affect the potential of nES cells to attain the TE programme. Specifically, the polycomb repressive complex 2 (PRC2) restricts the trophoblast fate during both hiTS cell and blastoid induction^25,26^. Despite this, additional barriers to acquiring the trophoblast fate, whether epigenetic or otherwise, are likely to exist.

In this study, we sought to explore the role of metabolism as a modulator of cell state transitions in the context of stem cell models of human pre-implantation development. Mouse studies revealed that early development is accompanied by a dramatic metabolic rewiring with oxygen consumption and tricarboxylic acid (TCA) cycle activity reaching their peak at the blastocyst stage^27,28^. Transcriptomic analysis predicts that this is broadly conserved across mammals, including humans^29^. Nevertheless, it remains unclear if there is metabolic asymmetry between the Epi and the TE of the developing human blastocyst. This is particularly important because not only do signalling and epigenetics regulate cell state transitions, but so does metabolism. Indeed, mouse naive pluripotency is stabilized through TCA cycle stimulation using α-ketoglutarate (αKG) supplementation^30,31^. Furthermore, differential glucose metabolism drives cell fate acquisition in the primitive streak during mouse gastrulation^32^. In addition to their function in energy and biomass production, molecules such as αKG and acetyl-coenzyme A (acetyl-CoA) are critical links between metabolism and the regulation of chromatin, as they are required for the activity of histone demethylases and histone acetyltransferases (HATs), respectively^30,33,34^. Metabolism not only regulates the stem cell epigenome but can also affect the activity of signalling pathways. In the mouse morula, Hippo signalling is modulated by metabolic regulation of YAP through glucose-dependent posttranslational modifications^35^. Despite these findings, little is known about the metabolic state of human nES cells or hiTS cells derived from them, and how their developmental potential may be modulated by metabolic regulation. Moreover, it remains unclear which regulatory pathways attune efficient TE maturation and its competence for implantation.

Here, we combine targeted metabolomics, transcriptomics and functional assays to uncover a considerable metabolic asymmetry between nES cells and hiTS cells, marked by high levels of αKG in the trophoblast lineage. This metabolic signature is coupled to cell fate choice, as TCA cycle stimulation by cell-permeable αKG increases competence of nES cells specifically towards the TE fate. We identify αKG-sensitive histone acetylation and the control of pluripotency as important links in regulating this process. In addition, increased αKG availability promotes cellular polarization and TE maturation during blastoid formation. Thus, our work indicates that a positive feedback loop might exist between TCA cycle regulation and the TE fate in early human development.

## Results

### Metabolic asymmetry between nES cells and hiTS cells regulates trophoblast induction

**Fig. 1:**
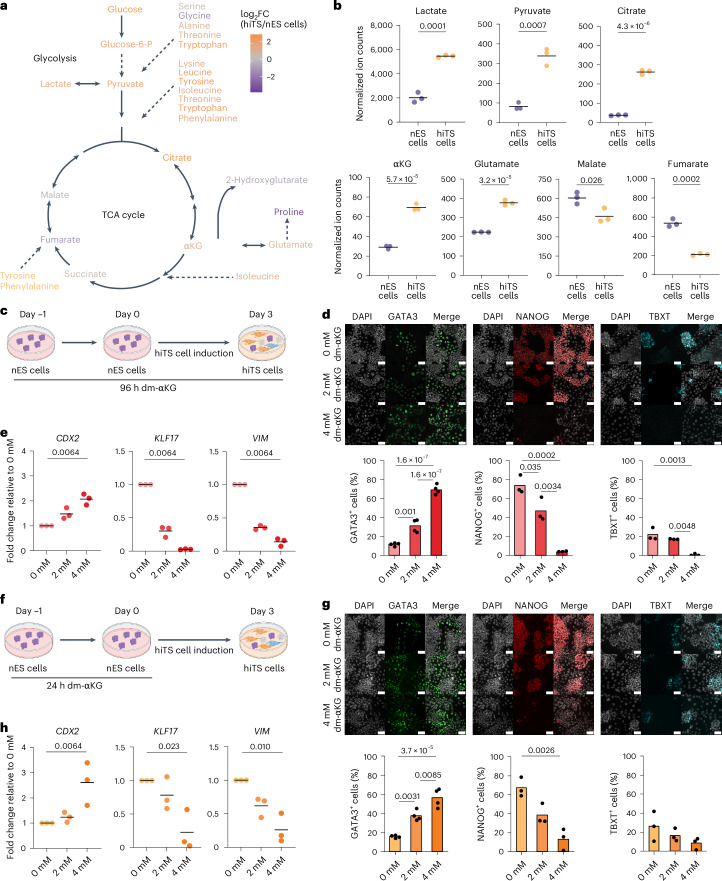
Metabolic asymmetry between nES cells and hiTS cells regulates trophoblast induction. **a**, A schematic representation of measured metabolites in glycolysis and TCA cycle; metabolites are colour coded on the basis of their log_2_ fold change (FC) between hiTS cells and nES cells. **b**, Ion counts of metabolites measured by gas chromatography–mass spectrometry normalized to protein content (the line shows the mean). **c**, A schematic of hiTS cell induction with 96 h dm-αKG treatment. **d**, Top: representative IF images of trophoblast (GATA3), pluripotency (NANOG) and primitive streak (TBXT) markers at day 3 of hiTS cell induction, treated for 96 h with 0, 2 or 4 mM of dm-αKG. Bottom: IF quantification. **e**, Expression levels of trophoblast (*CDX2*), pluripotency (*KLF17*) and extra-embryonic mesoderm (*VIM*) marker genes, determined by RT-qPCR. The expression values were normalized to *TBP* and presented as fold change relative to the untreated day 3 hiTS cells. **f**, A schematic of hiTS cell induction with 24 h dm-αKG pretreatment. **g**, Top: representative IF images of GATA3, NANOG and TBXT at day 3 of hiTS cell induction, treated for 24 h with 0, 2 or 4 mM of dm-αKG. Bottom: IF quantification. **h**, Expression levels of *CDX2*, *KLF17* and *VIM*, determined by RT-qPCR. The expression values were normalized to *TBP* and presented as fold change relative to the untreated day 3 hiTS cells. Data are presented as the mean and data points of *N* = 3 biological replicates (**b**, **d**, **e**, **g** and **h**). *P* values were calculated by two-sided unpaired *t*-tests (**b**), ordinary one-way analysis of variance (ANOVA) tests (**d** and **g**) or Kruskal–Wallis tests with Bonferroni correction (**e** and **h**). Scale bars, 50 μm. Created with BioRender.com (**c** and **f**). Source data

**Extended Data Fig. 1:**
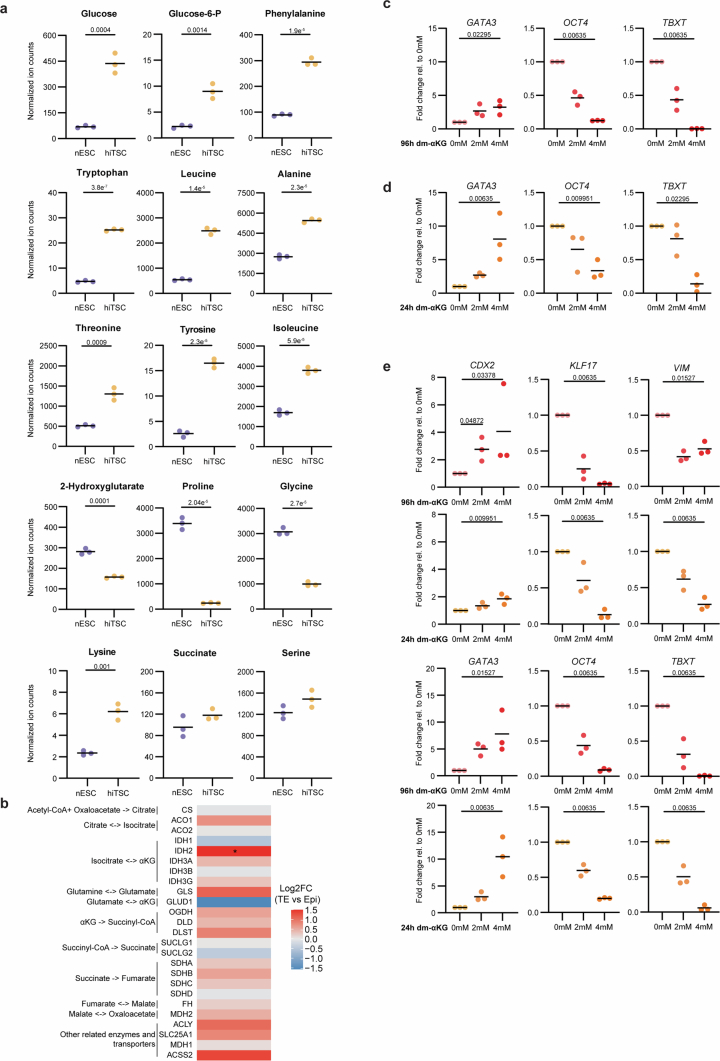
Metabolic asymmetry between nESC and hiTSC regulates trophoblast induction. **a**, Ion counts of metabolites measured by GC-MS normalised to protein content. nESC, naive embryonic stem cells; hiTSC, human induced trophoblast stem cells. **b**, Heatmap of Log2 fold change in the expression of key metabolic enzymes between in vivo TE and Epiblast. Median expression values were extracted from ref. ^10^. *IDH2 fold change is out of range and is 6.28. **c-d**, Expression levels of *GATA3*, *OCT4* and *TBXT* in d3 hiTSC measured by RT-qPCR. The expression values were normalised to *TBP* and presented as fold change relative to the untreated d3 hiTSC. Shown are samples with dm-αKG treatment for 96 h (**c**) or 24 h (**d**). **e**, Expression levels of *CDX2*, *GATA3*, *KLF17*, *OCT4, VIM* and *TBXT*, in HNES1 cells determined by RT-qPCR. The expression values were normalised to *TBP* and presented as fold change relative to the untreated d3 hiTSC. HNES1 were treated with dm-αKG for 96 h (red panels) or 24 h (yellow panels). Data is presented as the mean and data points of *N* = 3 biological replicates. *P* values were calculated by two-sided unpaired *t*-tests (**a**) or by Kruskal–Wallis test with Bonferroni correction (**c-e**). Source data

We set out to interrogate how metabolism regulates human TE and trophoblast induction and maturation. To gain insight into this question, we first performed targeted mass-spectrometry-based metabolomics to measure intracellular metabolite abundance in H9 nES cells cultured in tt2iLGö^12^ and in hiTS cells derived from them^20^. These two self-renewing cultures model the pre-implantation Epi and peri-implantation cytotrophoblast cells, respectively^36^. The targeted metabolites covered key nodes of central carbon metabolism, namely, glycolysis, the TCA cycle and amino acids. hiTS cells showed a significant increase in the concentration of glucose and glycolysis intermediates (glucose 6-phosphate, pyruvate, lactate and alanine) compared with nES cells (Fig. 1a,b and Extended Data Fig. 1a). This probably relates to increased expression of GLUT1, a key glucose transporter^20^. In addition to differences in glycolysis, TCA cycle intermediates displayed a notable asymmetry between the two models. The first half of the cycle was increased in hiTS cells (citrate, αKG and glutamate), while nES cells had increased concentrations in metabolites of the second half of the cycle, namely, fumarate and malate, that are located downstream of the connection between the TCA cycle and oxidative phosphorylation (Fig. 1a,b and Extended Data Fig. 1a). Of note, enzymes of this first half of the TCA cycle also translocate to the nucleus of mouse pre-implantation embryos and ES cells where they regulate histone methylation and acetylation^37,38^. Metabolites that act as anaplerotic substrates entering the TCA cycle in different points were also significantly increased in hiTS cells (Fig. 1a,b, Extended Data Fig. 1a). Overall, the analysis reveals the presence of a metabolic asymmetry in the TCA cycle between the hiTS cells and nES cells, with significant accumulation of αKG in hiTS cells. The latter metabolite is of interest because it can directly modulate the activity of multiple chromatin-modifying enzymes^39^. To assess whether metabolic rewiring also takes place during in vivo TE induction, we extracted the median expression of all TCA cycle enzymes from published single-cell RNA sequencing (scRNA-seq) datasets^10^. This revealed that most TCA cycle enzymes are upregulated in the TE (Extended Data Fig. 1b). Indeed, the TE from B3–B4 stage blastocysts upregulates genes relating to oxidative phosphorylation, and soon after, at B6 stage, it also increases the expression of amino-acid biosynthetic genes^40^. Furthermore, there is a strong activation of mitochondrial respiration genes in TE-like cells of the blastoid^21^. This metabolic state is also predicted to be further reprogrammed upon implantation^29^. All in all, there is striking metabolic asymmetry between the TE and Epi lineage in vitro and likely also in vivo. The potential functionality of this metabolic signature in regulating cell fate remains unclear.

**Extended Data Fig. 2:**
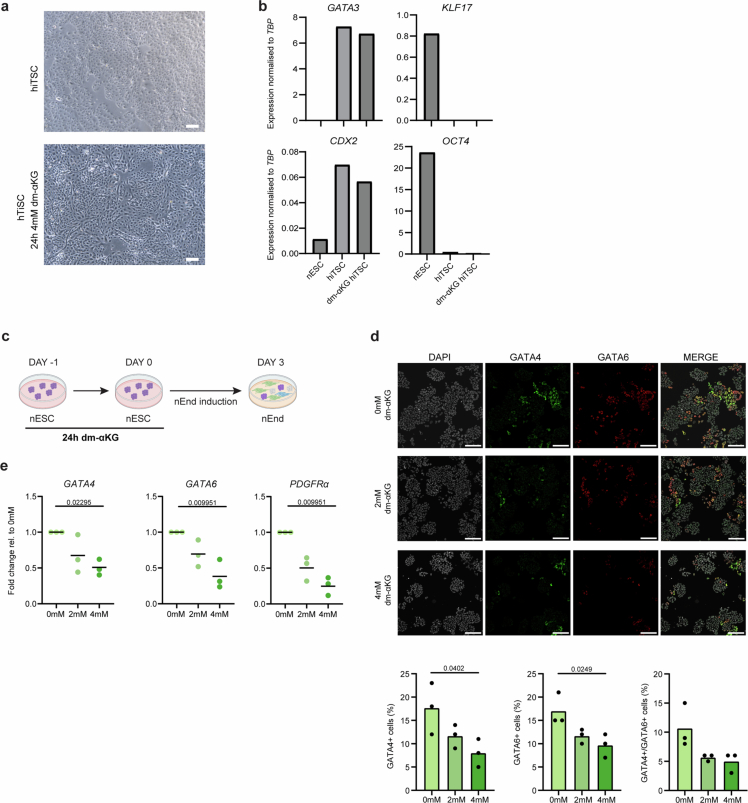
Dm-αKG treated nESC give rise to stable hiTSC line but have reduced competence towards primitive endoderm. **a**, Brightfield images of hiTSC stable cell lines ( > passage 5) derived from tt2iLGö nESC with or without 24 h 4mM dm-αKG pre-treatment. Scale bar = 200 μm. **b**, Expression levels of *CDX2*, *GATA3*, *KLF17* and *OCT4*, determined by RT-qPCR. The expression values were normalised to *TBP. N* = 1. **c**, Schematic of nEnd induction with 24h dm-αKG pre-treatment. **d**, Top panel: representative IF images of GATA4 and GATA6 at d3 of nEnd induction, treated for 24 h with 0, 2 or 4 mM of dm-αKG. Bottom panel: IF quantification. **e**, Expression levels of *GATA4*, *GATA6* and *PDGFRα*, determined by RT-qPCR. The expression values were normalised to *TBP* and presented as fold change relative to the untreated d3 nEnd. Data is presented as the mean and data points of *N* = 3 biological replicates (**d** and **e**). *P* values were calculated by ordinary one-way ANOVA tests (**d**) or Kruskal–Wallis tests with Bonferroni correction (**e**). Scale bar = 100 μm. (**c**) Created with BioRender.com. Source data

To gain mechanistic insights into the role of this metabolic asymmetry, we used both hiTS cell and blastoid models. First, we focused on the initial switch to TS cell identity, when the first GATA3-positive cells arise from nES cells (Fig. 1c). Under control conditions, only about 11% of cells express GATA3 by day 3, while other cells retain the pluripotency marker NANOG (Fig. 1d). Surprisingly, we found that about 20% of cells upregulate the primitive streak marker TBXT (Fig. 1d). This suggests that within the culture many nES cells might not only enter primed pluripotency but also initiate mesodermal differentiation. Because αKG levels were elevated in hiTS cells compared with nES cells, and αKG is known to regulate differentiation and chromatin in other models, we reasoned that this might be coupled to the trophoblast transcriptional programme^30,31,33^. To address if this is the case, we treated cells with dm-αKG, a cell-permeable form of αKG. A 96 h treatment with dm-αKG before and during hiTS cell derivation (Fig. 1c) resulted in a dose-dependent increase in the efficiency of GATA3 induction and a reciprocal suppression of NANOG and TBXT (Fig. 1d). Reverse transcription quantitative PCR (RT-qPCR) confirmed the upregulation of TS-cell markers (*CDX2* and *GATA3)* and reciprocal loss of markers of pluripotency (*OCT4* and *KLF17*), primitive streak (*TBXT*) and extra-embryonic mesoderm (*VIM*) (Fig. 1e and Extended Data Fig. 1c). Due to the strength and rapidity of the phenotype, we reasoned that dm-αKG could increase the competence of nES cells to induce GATA3 rather than acting during the cell fate transition. To address this, we shortened dm-αKG treatment to only 24 h before hiTS cell induction, when cells are still grown in naive tt2iLGö conditions (Fig. 1f). Immunofluorescence (IF) and RT-qPCR results revealed that such pretreatment led to a robust increase in TS-cell-marker gene expression (*GATA3* and *CDX2*) and reciprocal silencing of pluripotency-associated genes (*NANOG* and *OCT4*) (Fig. 1g,h and Extended Data Fig. 1d). Upon removing dm-αKG, these cultures successfully established a stable bona fide hiTS cell line (Extended Data Fig. 2a,b). We could further confirm these findings in another nES cell line HNES1 (ref. ^41^) (Extended Data Fig. 1e). We next investigated whether these effects are specific to the TE cell fate, or if dm-αKG treatment also enhances the competence of nES cells towards the other blastocyst lineage, namely, the primitive endoderm (PrE). To test this, we induced nES cells towards naive extra-embryonic endoderm (nEnd) for 3 days (Extended Data Fig. 2c)^16^. At this early stage, the first GATA4/GATA6 double-positive nEnd cells appear in approximately 10% of the population (Extended Data Fig. 2d). However, unlike during TS cell induction, dm-αKG pretreatment resulted in a reduced expression of PrE markers such as *GATA4*, *GATA6* and *PDGFRα* (Extended Data Fig. 2d,e). Collectively, these findings suggest that elevated intracellular levels of αKG in nES cells specifically enhance their competence towards TE-cell-like GATA3-positive cells, but not towards PrE-like cells or off-target fates such as the primitive streak or extra-embryonic mesoderm.

### Metabolic rewiring by dm-αKG increases nES cell competence towards the TE fate

**Fig. 2:**
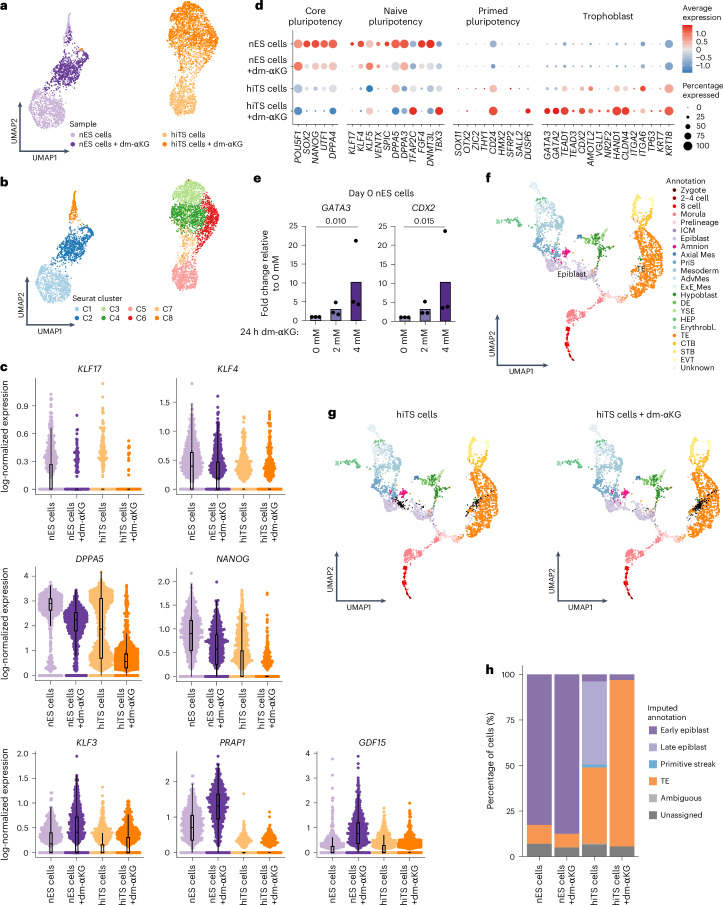
Metabolic rewiring by dm-αKG increases nES cell competence towards the TE fate. **a**,**b**, UMAP plots from scRNA-seq analysis of samples collected as in Fig. 1f. Cells are coloured according to sample (**a**) or graph-based clusters (C1–C8, **b**) (*N* = 1 experiment, *n* = 5,346 cells). **c**, Combined beeswarm and box plots of log-normalized gene expression values of pluripotency markers (*KLF17*, *KLF4*, *NANOG* and *DPPA5*) and early Epi markers (*KLF3*, *PRAP1* and *GDF15*). The sample type is shown with single cells plotted as dots; minima and maxima of the box plots span the interquartile range (IQR) 75th percentile subtracted from the 25th percentile, the centre indicates the median, and the whiskers extend to values within 1.5 times the IQR (*N* = 1 biological replicate, nES cells *n* = 1.158, nES cells + dm-αKG *n* = 900, hiTS cells *n* = 1,577, hiTS cells + dm-αKG *n* = 1,711) **d**, The expression of selected lineage-specific marker genes across samples. The size of the dots represents the proportion of cells in the sample expressing the given gene, and the colour encodes the scaled average expression. **e**, Levels of expression of trophoblast marker genes (*GATA3* and *CDX2*) as determined by RT-qPCR. The expression values were normalized to *TBP* and are presented as fold change relative to the untreated day 0 nES cells. Data are presented as the mean and data points of *N* = 3 biological replicates. **f**, A 2D UMAP visualization of integrated scRNA-seq data derived from human pre-implantation and post-implantation embryos with annotation from ref. ^43^. ICM, inner cell mass; Axial Mes, axial mesoderm; PriS, primitive streak; AdvMes, advanced mesoderm; ExE_Mes, extra-embryonic mesoderm; DE, definitive endoderm; YSE, yolk sac endoderm; HEP, hemato-endothelial progenitors; Erythrobl., erythroblasts; TE, trophectoderm; CTB, cytotrophoblast; STB, syncytiotrophoblast; EVT, extravillous trophoblast. **g**, In vivo reference data with in vitro day 3 hiTS cells, generated from control or dm-αKG-treated nES cells. The black dots show neighbourhoods of in vitro generated cells projected onto a reference UMAP. **h**, Imputed annotation of in vitro samples from reference in vivo dataset. Unassigned and ambiguous labels refer to cells with either none or with more than two imputed annotations, respectively. *P* values were calculated by Kruskal–Wallis tests (**e**) with Bonferroni correction. Source data

**Extended Data Fig. 3:**
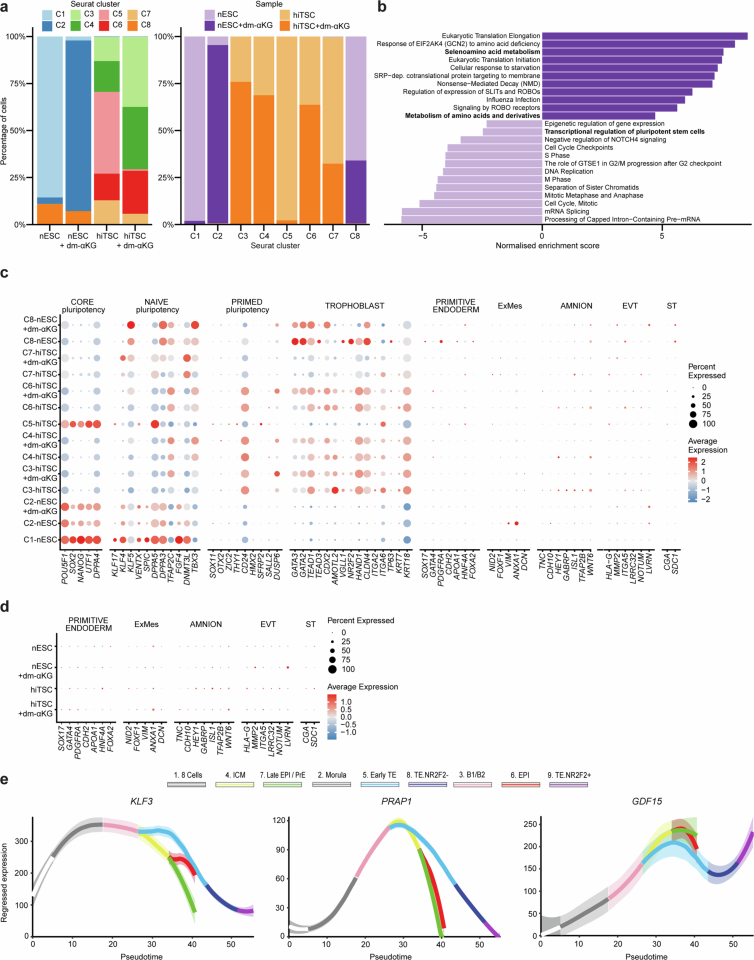
Metabolic rewiring by dm-αKG alters the competence of nESC towards the TE fate. **a**, Bar charts showing the percentage of cells from different Seurat clusters in each sample (left); and how samples contribute to Seurat clusters (right). **b**, Gene set enrichment analysis for differentially expressed genes between Seurat cluster C1 and C2. Shown are some significantly enriched Reactome terms. Normalised enrichment score is positive when terms are enriched in genes upregulated in C2 vs C1. **c**, Expression of selected lineage-specific marker genes across Seurat clusters separated according to sample type. Combinations of clusters and samples containing less than 20 cells are not shown. Dot size represents the proportion of cells in which the gene was detected, and colour indicates scaled average expression. **d**, Expression of selected lineage-specific marker genes across samples related to Fig. 2d. The size of the circles represents the proportion of cells in the sample with the indicated gene expression level. **e**, Pseudotime plots showing expression dynamics of putative early epiblast/ICM markers (*KLF3, PRAP1, GDF15*) also upregulated within Seurat cluster C2. Data was extracted from ref. ^43^. ICM: inner cell mass; EPI: epiblast; PrE: primitive endoderm; TE: trophectoderm; B1/B2: early blastocyst at B1/B2 stage. Source data

To address how αKG can increase competence of nES cells, we focused on heterogeneities within the 24 h pretreatment experimental design (Fig. 1f). To this end, we have performed scRNA-seq in nES cells treated or untreated for 24 h with 4mM dm-αKG and in day 3 hiTS cells induced from these two conditions. Uniform manifold approximation and projection (UMAP) analysis revealed that hiTS cell samples are well separated from nES cells (Fig. 2a). Subsequent graph-based clustering identified eight clusters where C1 and C2 are populated mainly with control and dm-αKG-treated nES cells, respectively (Fig. 2a,b and Extended Data Fig. 3a). C8 is populated by both nES cell samples and consists of rare spontaneously arising GATA3-positive cells^26^. Meanwhile, the separation between hiTS cell samples was less prominent with clusters C3, C4, C6 and C7 being populated by both control hiTS cells and those pretreated with dm-αKG (Fig. 2a,b and Extended Data Fig. 3a). Nevertheless, C5 was almost exclusively composed of control hiTS cell sample. We first focused on nES cell samples and found that dm-αKG treatment leads to a significant attenuation of expression of multiple pluripotency genes (including *KLF17*, *KLF4*, *NANOG* and *DPPA5*) (Fig. 2c,d and Extended Data Fig. 3c). Among the top upregulated genes, there were three that are typically expressed in the early Epi (*KLF3*, *PRAP1* and *GDF15*) (Fig. 2c and Extended Data Fig. 3e). Gene set enrichment analysis (GSEA) of differentially expressed genes confirmed that, upon dm-αKG treatment, downregulated genes are enriched for pluripotency terms along with others (Extended Data Fig. 3b and Supplementary Table 1). Conversely, genes upregulated in nES cells treated with dm-αKG were enriched for, among others, metabolic pathways (Extended Data Fig. 3b and Supplementary Table 1), indicating that αKG supplementation could lead to metabolic rewiring. It is interesting that scRNA-seq did not detect the upregulation of typical TE or trophoblast markers in dm-αKG-treated nES cells (Fig. 2c,d). Nevertheless, a more sensitive RT-qPCR analysis found that there is significant derepression of TE and trophoblast markers (*GATA3* and *CDX2*) in dm-αKG-treated nES cells (Fig. 2e). All in all, dm-αKG treatment of nES cells under tt2iLGö culture conditions attenuates the expression of pluripotency genes.

**Extended Data Fig. 4:**
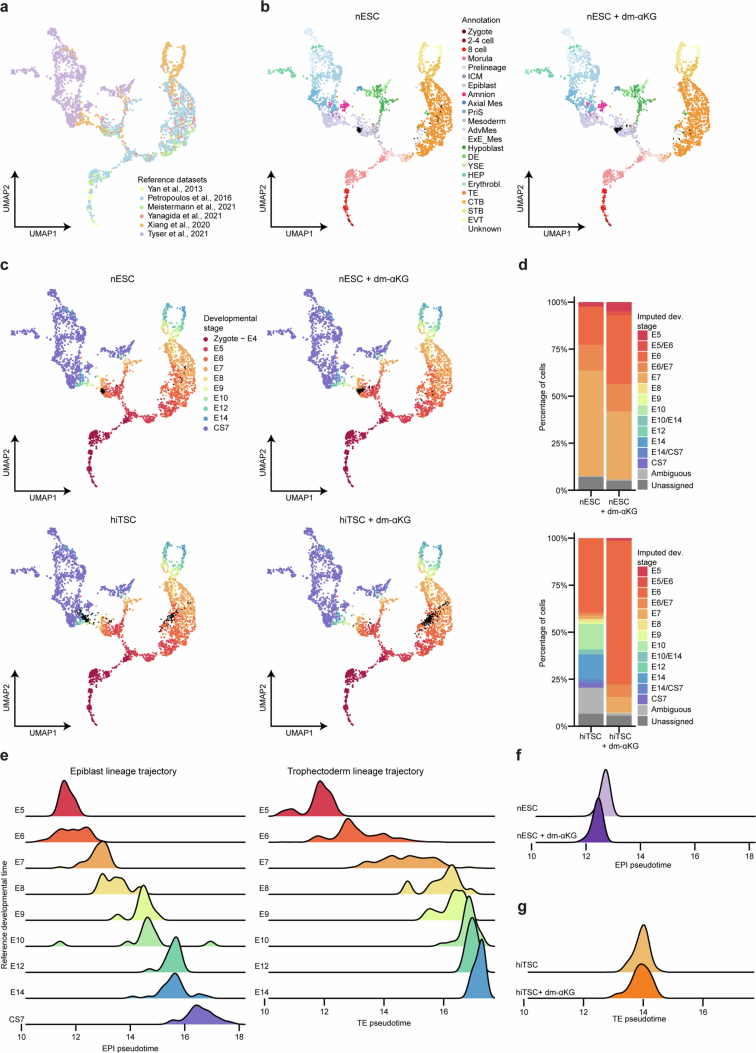
Metabolic rewiring by dm-αKG allows for efficient TE-like cell induction. **a**, UMAP of human pre-implantation and post-implantation embryos with annotation for scRNA-seq data integration from ref. ^43^ with cells coloured by reference dataset of origin. **b-c**, Projection of in vivo reference data with in vitro d0 nESC or d3 hiTSC, control or pre-treated with dm-αKG for 24 h. Black dots show neighbourhoods of in vitro generated cells projected onto a reference UMAP. Colour code represents different annotations (**b** – as in Fig. 2g) or developmental time (**c**). **d**, Imputed developmental time of in vitro samples from reference in vivo dataset. E: embryonic day, CS: Carnegie Stage. Unassigned and ambiguous labels refer to cells with either none or with more than two imputed stages respectively. **e**, Reference pseudotime trajectories of the in vivo epiblast (EPI: left) and trophectoderm (TE: right) related to the reference developmental time based on ref. ^43^. **f-g**, Distribution of pseudotime values in nESC (**f**) and d3 hiTSC (**g**) cells which are related to the in vivo EPI and TE respectively. Pseudotime trajectories were identified from in vivo reference (**e**). Source data

When focusing on the day 3 hiTS cell samples, we found that dm-αKG pretreatment led to more homogeneous populations than untreated hiTS cells. Indeed, there was a striking upregulation of TE and trophoblast marker genes (for example, *GATA3*, *CDX2* and *NR2F2*), with lowered expression of pluripotency genes (for example, *DPPA5* and *NANOG*) (Fig. 2c,e and Extended Data Fig. 3c), confirming previous IF and RT-qPCR results (Fig. 1g,h). Furthermore, we did not detect markers of other possible off-target populations such as the amnion or extra-embryonic mesoderm in either sample (for example, *TNC* and *VIM*) (Extended Data Fig. 3d)^42,43^. This confirmed that dm-αKG treatment allows robust activation of the TE transcriptional programme. To further verify if metabolic treatment aids in the establishment of TE-like cells, we have mapped our scRNA-seq onto a stable reference UMAP of human early development (Fig. 2f and Extended Data Fig. 4a)^9,10,22,43–46^. This analysis confirmed that 91.4% of cells from the hiTS cell + 24 h dm-αKG sample were closely related to in vivo TE and predominantly at embryonic day (E) 6–7 pre-implantation blastocyst stage (Fig. 2g,h and Extended Data Fig. 4c,d). By contrast, only 41.9% of control hiTS cells were annotated as TE, with many cells remaining in the late Epi state, which we define as Epi starting from E9 onwards (Fig. 2g,h and Extended Data Fig. 4c,d). This confirmed that dm-αKG pretreatment allowed for nearly homogeneous induction of a transcriptional network reminiscent of in vivo TE. Furthermore, under control conditions, a large subset of cells becomes erroneously primed towards post-implantation Epi and even upregulates the primitive streak marker *TBXT* (Fig. 1d). While focusing on the nES cell samples, we found that both control and dm-αKG-treated cells were predominantly related to pre-implantation early Epi (82.6% and 87.6%, respectively) (Fig. 2h, Extended Data Fig. 4b). Furthermore, the dm-αKG treatment resulted in a higher percentage of cells relating to E6 developmental stage or earlier (43.7% versus 22.6% in controls) (Extended Data Fig. 4c,d). Pseudotime trajectory analysis confirmed an earlier developmental phenotype in nES cells treated with dm-αKG (Extended Data Fig. 4e–g). This result is in line with reduced expression of pluripotency genes, which in vivo still increase while the Epi matures from E6 to E7–8. All in all, dm-αKG treatment leads to an attenuated pluripotency network, paving the way for a more efficient induction of TE-like cells. While metabolic intervention alone fell short of triggering a cell fate transition in nES cells, it did enhance the competence of these cells specifically towards the TE fate. This observation suggests that an increase in αKG plays a permissive role, rather than an inductive one, in specifying the trophoblast lineage, that is, it permits cells to efficiently respond to specific signalling cues towards the TE fate. Our data also indicate that, while hiTS cells are a model of peri-implantation trophoblast, at early stages of their induction, cells transit through a pre-implantation TE-like state.

### Metabolically induced hypoacetylation increases nES cell competence towards TE

**Fig. 3:**
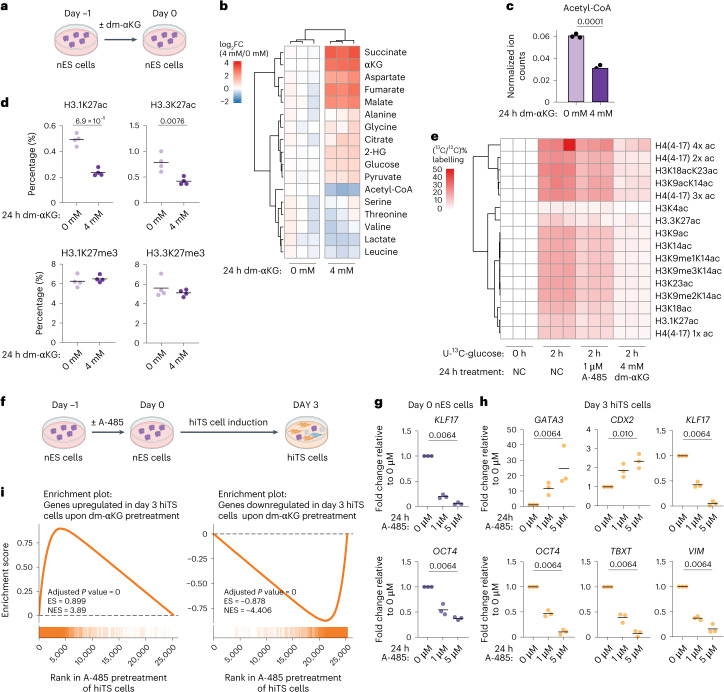
Metabolically induced hypoacetylation increases nES cell competence towards TE. **a**, A schematic of nES cell treatment with dm-αKG for 24 h. **b**, A heat map showing log_2_ fold change (FC) in normalized ion counts between 4 mM and 0 mM dm-αKG samples. FC was computed on the basis of the average across 0 mM samples. 2-HG, 2-hydroxyglutarate. *N* = 3 biological replicates. **c**, Ion counts of acetyl-CoA measured by liquid chromatography–mass spectrometry normalized to protein content (the bar shows the mean). *N* = 3 biological replicates. **d**, Global histone PTM levels quantified by mass spectrometry. *N* = 4 biological replicates. **e**, Mass spectrometry quantification of newly deposited histone acetylation using U-^13^C-glucose labelling. The scale shows the percentage of ^13^C-labelled acetyl-peptides relative to unlabelled ^12^C acetyl-peptides. NC, negative control or no treatment. *N* = 3 biological replicates. **f**, A schematic of hiTS cell induction with 24 h of P300i (A-485) treatment. **g**,**h**, Effects of P300 inhibition on expression levels measured by RT-qPCR. The data are normalized to *TBP* and presented as fold change to untreated control for nES cells (**g**) and day 3 hiTS cells (**h**). Shown are markers of the trophoblast (*GATA3* and *CDX2*), pluripotent cells (*KLF17* and *OCT4*), primitive streak (*TBXT*) and extra-embryonic mesoderm (*VIM*). Data are presented as the mean and data points of *N* = 3 biological replicates. **i**, Enrichment plots of gene sets defined by genes significantly upregulated (left) or downregulated (right) in day 3 hiTS cells upon dm-αKG pretreatment. Along the *x* axis, genes are ranked by Wald statistic (from positive to negative) from DESeq2 for P300i (A-485) pretreatment compared with untreated control. Vertical lines indicate where the members of the gene set appear in the ranked list of genes. The curve shows the running enrichment score (ES), and it peaks at the ES for the given gene set. NES, normalized enrichment score. *P* values were calculated by two-sided unpaired *t*-tests (**b**–**d**), one-way Kruskal–Wallis tests with Bonferroni correction (**g** and **h**) or two-sided fast pre-ranked gene set enrichment analysis (FGSEA) with Benjamini–Hochberg correction (**i**). Created with BioRender.com (**a**). Source data

**Extended Data Fig. 5:**
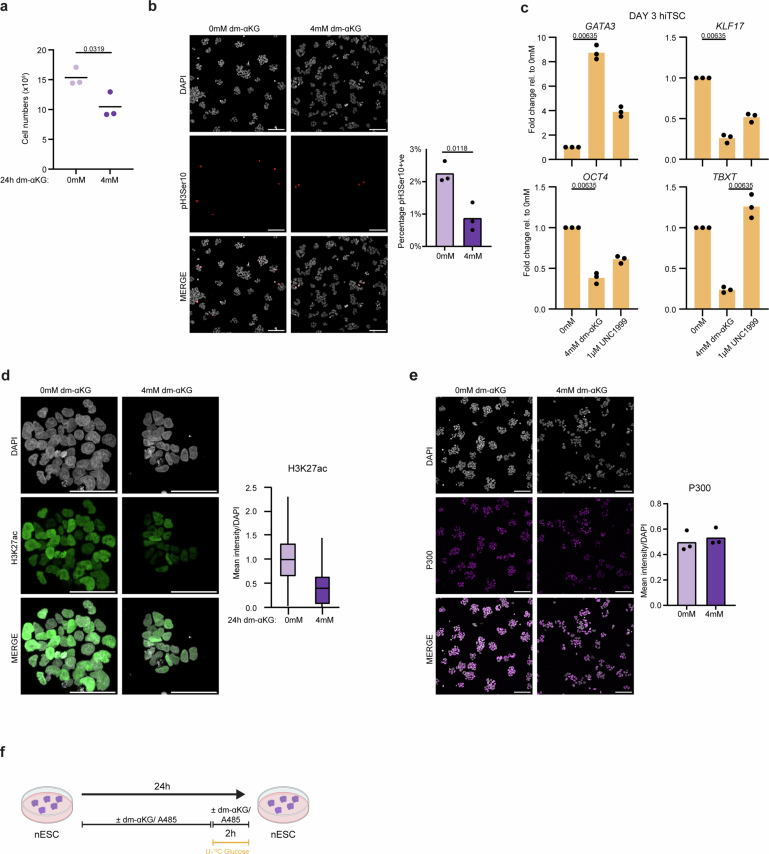
Treatment of nESC by dm-αKG leads to histone hypoacetylation. **a**, Cell number quantification of nESC treated with 0 or 4 mM of dm-αKG. Data is presented as the mean and data points of *N* = 3 biological replicates. 1.6 ×10^4^ cells per cm2 were seeded in a 6-well plate pre-coated with ECMatrix™-511 Silk E8 Laminin Substrate. nESC were cultured for 48 h before treatment with 0 or 4 mM of dm-αKG for 24 h. Two 6-well wells were collected per condition for each biological replicate. **b**, Left panel: representative IF images of Phospho-Histone H3-Ser10 (pH3S10) in nESC treated with 0 or 4 mM of dm-αKG for 24 h. Right panel: IF quantification showing the percentage of pH3Ser10+ve cells. Data is presented as the mean and data points of *N* = 3 biological replicates. Scale bar = 100 μm. **c**, Expression levels of *GATA3*, *KLF17*, *OCT4* and *TBXT*, in d3 hiTSC determined by RT-qPCR. The expression values were normalised to *TBP* and presented as fold change relative to the untreated d3 hiTSC. Naive ESC were treated with either 4mM dm-αKG or 1 µM UNC1999 for 24 h prior to being induced without treatment towards hiTSC. Data is presented as the mean and data points of *N* = 3 biological replicates. **d**, Left panel: representative IF images of H3K27ac at d0 in nESC treated with 0 or 4 mM of dm-αKG. Right panel: IF quantification showing H3K27ac intensity normalised to DAPI. *N* = 1 biological replicate, nESC n = 391, nESC + 4mM dm-αKG n = 468. Box plot shows: center line: median; box minima and maxima: 25th and 75th percentiles; whiskers: 1.5x interquartile range, 75^th^ percentiles subtracted from 25^th^ percentiles. Scale bar = 50 μm. **e**, Left panel: representative IF images of P300 at d0 in nESC treated with 0 or 4 mM of dm-αKG. Right panel: IF quantification showing P300 intensity normalised to DAPI. *N* = 3 biological replicates. Scale bar = 100 μm. **f**. Experimental set-up of U-^13^C-Glucose labelling in nESC treated with 0 or 4 mM of dm-αKG or 1 mM A-485. *P* values were calculated by Kruskal–Wallis test with Bonferroni correction (**c**) or two-sided unpaired *t*-test (**a, b, e**). (**f**) Created with BioRender.com. Source data

To investigate the mechanisms by which dm-αKG increases the competence of nES cells towards TE-like cells, we first analysed whether dm-αKG induces metabolic rewiring of nES cells. To address this, we quantified intracellular metabolite levels in nES cells treated with 4 mM dm-αKG for 24 h (Fig. 3a). As expected, we found that dm-αKG supplementation significantly increased the abundance of intracellular αKG, as well as the levels of all TCA cycle intermediates (Fig. 3b). While this might lead to altered bioenergetic state, dm-αKG did not increase cell proliferation; rather, there was a decrease in the percentage of mitotic cells (Extended Data Fig. 5a,b). Apart from the TCA cycle effects, glucose and pyruvate were also significantly elevated. Interestingly, we observed pyruvate routing towards downstream metabolites to be limiting, as evident by the decreased abundance of both lactate and acetyl-CoA (Fig. 3b,c). The latter is in agreement with the increased gene expression of pyruvate dehydrogenase lipoamide kinase isozyme 1 (PDK1) upon dm-αKG supplementation, a kinase that phosphorylates and inactivates the E1a subunit of the pyruvate dehydrogenase complex (PDH), which catalyses the conversion of pyruvate to acetyl-CoA^47^. Taken together, these data show that dm-αKG induces a striking rewiring of the core central carbon metabolism.

Metabolites of the TCA cycle are not only involved in energy and biomass production but can also directly affect the activity of signalling pathways and chromatin modifiers^39^. Indeed, αKG is directly involved in histone and DNA demethylation^30,33^. To test the impact of dm-αKG on chromatin modifications, we have performed global histone posttranslational modification quantification using mass spectrometry in nES cells treated for 0 h or 24 h with dm-αKG (Fig. 3a). Surprisingly, this revealed that the metabolic treatment has little effect on the levels of histone methylation (Fig. 3d and Supplementary Table 2). Specifically, the global levels of H3K27me3, a known barrier for trophoblast specification in human, do not change upon dm-αKG treatment (Fig. 3d)^25,26^. While we did not observe global loss of the H3K27me3, it remains possible that local demethylation might take place. Therefore, we compared the phenotype of day 3 hiTS cells induced from nES cells pretreated with either dm-αKG or a PRC2 inhibitor (1 µM UNC1999). These data revealed that the phenotype of reduced pluripotency markers (*KLF17* and *OCT4*) and reciprocal induction of *GATA3* is less striking in PRC2i compared with dm-αKG (Extended Data Fig. 5c). Importantly, unlike PRC2i, dm-αKG pretreatment resulted in the downregulation of primitive streak marker *TBXT*. Together this indicates that PRC2i and dm-αKG lead to distinct phenotypes; thus, the remodelling of H3K27me3 is unlikely to be the primary mechanism of action for increased αKG levels. Meanwhile, we found that αKG supplementation leads to reduced acetylation at many histone peptides, including at H3K27 (Fig. 3d and Supplementary Table 2), an effect further validated using IF (Extended Data Fig. 5d). Because our findings revealed limited effect on global histone methylation, and a phenotype distinct from that of PRC2i, we propose that under tested conditions the αKG concentration might not be rate limiting for histone demethylase activity. All in all, dm-αKG treatment in nES cells resulted in rapid global reduction of acetylation, especially at H3 lysine 27 with little change to global histone methylation levels.

We next asked about the mechanism leading to such global hypoacetylation. According to the differential gene expression analysis, dm-αKG does not result in significant upregulation of histone deacetylases or downregulation of HATs (Supplementary Table 3). Instead, the observed histone hypoacetylation could be a metabolic effect. Indeed, dm-αKG diminishes acetyl-CoA availability (Fig. 3c), which is required for HAT activity^34^. Alternatively, a stimulated TCA cycle could alter nicotinamide adenine dinucleotide (NAD) and nicotinamide metabolism, thus promoting deacetylation by sirtuins. To distinguish between these two possibilities, we performed metabolic labelling of newly deposited acetylation marks^48^. For this, nES cells were treated with either 0 mM or 4 mM dm-αKG. As a control, we also included a moderate inhibition of P300 (A-485, 1 μM), a key HAT that is stably expressed in our experiments (Extended Data Fig. 5e). After 22 h of treatment, cells were grown for 2 h with media containing uniformly labelled ^13^C-glucose (U-^13^C-glucose) (Extended Data Fig. 5f). In this way, newly deposited acetyl groups become detectable in mass spectrometry owing to their ^13^C labelling. We found that 2 h in U-^13^C-glucose media resulted in labelling of 4–38% of acetylation at specific histone peptides (Fig. 3e and Supplementary Table 4). Meanwhile, P300 inhibition led to decreased labelling at most peptides, thus validating our approach^49^. Strikingly, the addition of dm-αKG led to a significant depletion in the deposition of new acetylation marks (Fig. 3e and Supplementary Table 4). Together, this indicates that dm-αKG not only diminishes acetyl-CoA but also leads to reduced HAT activity.

**Extended Data Fig. 6:**
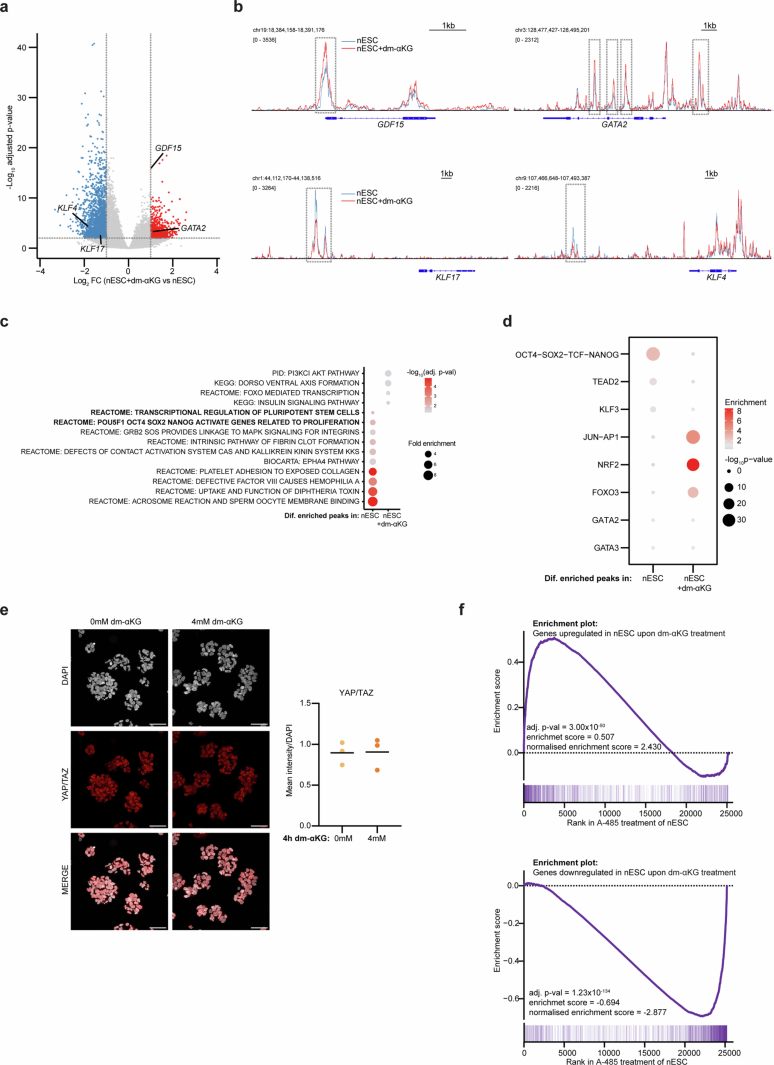
Treatment of nESC by dm-αKG alters enhancer usage. **a**, Differential peak enrichment analysis of ATAC-seq (N = 3 replicates). Peaks enriched in control and dm-αKG treated nESC are highlighted in blue and red respectively; the number of differentially enriched peaks in both conditions are indicated. Dashed lines indicate adjusted p-value < 0.01 and |Log2(FC) | > 1. **b**, Genome browser tracks displaying ATAC-seq signal across loci that are more (*GDF15, GATA2*) or less (*KLF17, KLF4*) accessible upon dm-αKG treatment of nESCs. Signals from control and dm-αKG-treated nESCs are shown in blue and red, respectively. Gray boxes highlight peaks with altered accessibility. **c**, GREAT gene set enrichment analysis of differentially enriched peaks is shown with fold enrichment plotted as dot size and adjusted p-value in colour scale. Peaks enriched in nESC + dm-αKG and in nESC compared to all peaks are shown. **d**, Transcription factor motif enrichment analysis on differentially enriched peaks is plotted. Dot colour represents enrichment relative to all peaks and dot size is -log_10_p-value. Motifs were grouped based on similarity and only one TF per related motif is shown. **e**, Left panel: representative IF images of YAP/TAZ at d0 in nESC treated with 0 or 4 mM of dm-αKG. Right panel: IF quantification showing YAP/TAZ intensity normalised to DAPI. *N* = 3 biological replicates. Scale bar = 20 μm. **f**, Enrichment plots of gene sets defined by genes significantly upregulated (top) or downregulated (bottom) in nESC upon 24h dm-αKG treatment. Along the x-axis genes are ranked by Wald statistic (from positive to negative) from DESeq2 for P300i (A-485) pre-treatment compared to untreated control. Vertical lines indicate where the members of the gene set appear in the ranked list of genes. The curve shows the running enrichment score (ES) and it peaks at the ES for the given gene set. *P* values were calculated by two-sided unpaired *t*-test (**e**) or two-sided FGSEA with Benjamini-Hochberg correction (**f**). Source data

Histone acetylation is predominantly deposited at gene regulatory elements (GRE) and can promote their activation^50–52^. To test how dm-αKG affects the GRE usage, we performed assay for transposase-accessible chromatin using sequencing (ATAC-seq) in nES cells. This revealed a significant but specific closing of some GREs upon dm-αKG treatment, with 2,479 and 793 elements becoming significantly less and more accessible, respectively (Extended Data Fig. 6a,b, adjusted *P* value <0.01; |log_2_FC| >1). In line with transcriptomic analysis, such closing GREs were enriched in proximity of OCT4- and SOX2-regulated genes (Extended Data Fig. 6c). Using motif enrichment analysis, we further identified putative transcription factors with altered activity upon dm-αKG treatment. Consistently with transcriptomic data, GREs becoming less accessible are enriched for OCT–SOX motifs and there is also low-level depletion of KLF and TEAD motifs (Extended Data Fig. 6d). Meanwhile, GREs becoming more open are enriched for JUN and FOS as well as FOX and NRF2 motifs (Extended Data Fig. 6d). Together this indicates that dm-αKG treatment leads to an attenuated pluripotency GRN and activation of the JUN–FOS signature, which is enriched in hiTS cell-like cells^53^. However, dm-αKG alone is not sufficient to robustly activate the TEAD–YAP or GATA2–GATA3 regulatory networks, which are the master regulators of TE fate. In line with this, we did not observe an increased nuclear YAP–TAZ staining upon dm-αKG treatment (Extended Data Fig. 6e) or opening of GREs with GATA motifs (Extended Data Fig. 6d). Moreover, TEAD motifs are already robustly enriched in nES cells in accordance with high basal levels of nuclear YAP and TAZ and its role in stem cell maintenance^5^. Thus, the metabolic rewiring induced by dm-αKG treatment not only results in transcriptional changes but also affects histone acetylation levels and alters GRE usage. Nevertheless, in line with the transcriptomic analysis, dm-αKG alone is not sufficient to change cell fate. As such, this metabolic switch does not function as an inductive signal; rather, its role is permissive, meaning that it permits cells to efficiently respond to specific signalling cues towards the TE fate.

One of the major phenotypes of dm-αKG treatment was the attenuation of the naive pluripotency network accompanied by metabolic regulation of HATs and histone hypoacetylation. However, metabolism has a plethora of cellular functions and it remains unclear how relevant this reduced acetylation is to increased nES cell competence towards the TE fate. To test if deacetylation alone leads to the attenuated pluripotency GRN, we performed a 24 h inhibition of the main H3K27 acetyltransferase P300 using a small molecule A-485 (Fig. 3f). Indeed, P300 inhibition resulted in robust and dose-dependent repression of pluripotency genes (*KLF17* and *OCT4*) (Fig. 3g). We further found that, just like dm-αKG, P300 inhibition increases the competence of nES cell induction towards the TE fate. At day 3, the hiTS cells originating from A-485 pretreated nES cells showed robust increase in TE-marker gene expression (*GATA3* and *CDX2*) and reciprocal repression in markers of pluripotency (*OCT4* and *KLF17*), primitive streak (*TBXT*) and extra-embryonic mesoderm (*VIM*) (Fig. 3h). Further mRNA-seq analysis confirmed that there is significant overlap in the transcriptional phenotype of increasing intracellular αKG levels and inhibiting P300. In nES cells, both upregulated and downregulated genes upon dm-αKG treatment showed a significant co-enrichment with those differentially expressed upon P300 inhibition (Extended Data Fig. 6f). Of note, P300i treatment results in a more severe transcriptional change in nES cells than that compared with dm-αKG treatment. Nevertheless, both drug treatments lead to a strikingly similar phenotype when nES cells are induced towards hiTS cells (Fig. 3i). Analysis of both upregulated and downregulated genes found that the co-enrichment is so high that it prevents the calculation of an exact *P* value (Fig. 3i). Together this indicates that reduced histone acetylation is sufficient to phenocopy dm-αKG treatment, but it is unclear if it is also necessary. All in all, we find that hypoacetylation leads to a similar phenotype of dm-αKG treatment that is the attenuation of pluripotency genes, thus diminishing the barrier towards TE induction.

### Increased αKG levels aid in aggregate polarization during blastoid induction

**Fig. 4:**
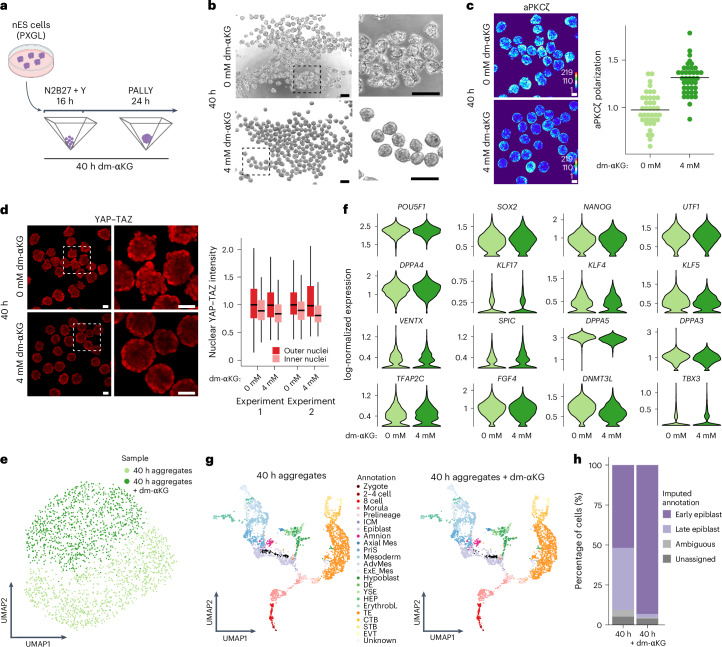
Increased αKG levels aid in polarization. **a**, A schematic of aggregate formation with 40 h dm-αKG treatment. **b**, Representative brightfield images of 40 h aggregates with or without dm-αKG treatment. Scale bars, 200 μm. *N* = 3 biological replicates. **c**, Representative IF images and analysis of a polarity marker aPKCζ in 40 h aggregates with or without dm-αKG treatment. IF quantification shows aPKCζ intensity within the apical domain normalized to the signal in the remaining part of the aggregate. Each dot represents one aggregate, *n* = 39–40 from *N* = 2 biological replicates; the line is the median. Scale bars, 50 μm. **d**, Representative IF images and analysis of YAP and TAZ in 40 h aggregates with or without dm-αKG treatment. Nuclear YAP and TAZ intensity is quantified in the outer and inner cells. The box plots show: centre line, median; box limits, 25th and 75th percentiles; whiskers, 1.5× interquartile range; outliers not shown. Scale bars, 50 μm. *N* = 2 biological replicates, EXP1 0 mM outer nuclei *n* = 363, EXP1 0 mM inner nuclei *n* = 316, EXP1 4 mM outer nuclei *n* = 580, EXP1 4 mM inner nuclei *n* = 256, EXP2 0 mM outer nuclei *n* = 480, EXP2 0 mM inner nuclei *n* = 214, EXP2 4 mM outer nuclei *n* = 477, EXP2 4 mM inner nuclei *n* = 216, minimum 18 aggregates quantified per experiment. **e**, UMAP plots from scRNA-seq analysis of samples collected as in Fig. 4a. Cells are coloured according to sample (*N* = 1 experiment, minimum 100 aggregates, *n* = 2,255 cells). **f**, Violin plots showing log-normalized expression of core and naive pluripotency markers in 40 h aggregates with or without dm-αKG treatment. **g**, Integration of in vivo reference data (Fig. 2f) with in vitro 40 h control or dm-αKG-treated aggregates. The black dots show neighbourhoods of in vitro generated cells projected onto a reference UMAP. ICM, inner cell mass; Axial Mes, axial mesoderm; PriS, primitive streak; AdvMes, advanced mesoderm; ExE_Mes, extra-embryonic mesoderm; DE, definitive endoderm; YSE, yolk sac endoderm; HEP, hemato-endothelial progenitors; Erythrobl., erythroblasts; TE, trophectoderm; CTB, cytotrophoblast; STB, syncytiotrophoblast; EVT, extravillous trophoblast. **h**, Imputed annotation of in vitro samples from reference in vivo dataset. Unassigned and ambiguous labels refer to cells with either none or with more than two imputed annotations, respectively. Created with BioRender.com (**a**). Source data

To test if the effect of high αKG levels during two-dimensional (2D) hiTS cell induction is recapitulated in a more physiological three-dimensional (3D) context, we performed experiments using the human blastoid model^21^ (Fig. 4a). In this case, we aggregated nES cells (PXGL culture conditions: PD0325901 (1 μM), XAV939 (2 μM), Gö 6983 (2 μM) and hLIF (10 ng ml^−1^)^54^) in AggreWells in the presence or absence of dm-αKG. Within the first 40 h of dm-αKG treatment, the aggregates were markedly more compacted than the controls (Fig. 4b), indicating a possible effect on the first TE specification step, which is apical polarization^2,21,55^. Indeed, dm-αKG treatment resulted in aggregates with increased aPKCζ polarization (Fig. 4c). Specifically, most of the control aggregates failed to downregulate aPKCζ within the inner cells, while dm-αKG addition resulted in robust aPKCζ enrichment but only on the apical domain of outer cells. Apical polarization allows the accumulation of nuclear YAP and TAZ in the outer cells, thus initiating TE specification^2,55^. In line with this, dm-αKG-treated aggregates showed a consistent nuclear YAP and TAZ within the outer polarized cells and allowed a more robust downregulation of YAP and TAZ within the inner cells destined for the ICM and Epi fate (Fig. 4d). This could indicate that metabolic treatment leads to a quicker or more synchronous induction of polarization, thus allowing regionalized YAP and TAZ nuclear activation.

**Extended Data Fig. 7:**
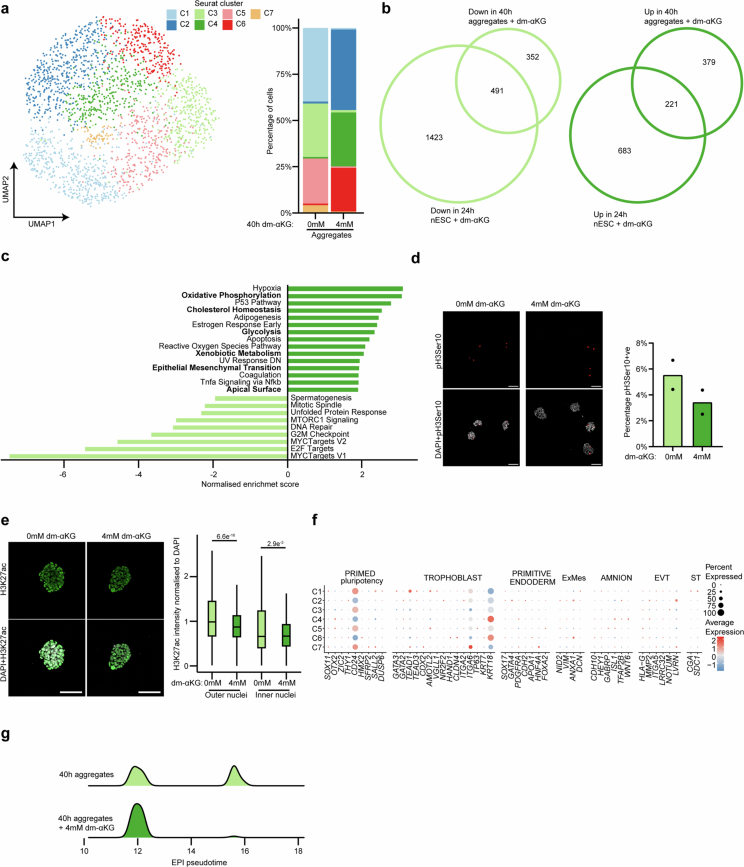
Treatment of 40 h aggregates with dm-αKG leads to improved apical polarisation. **a**, Left panel: UMAP plots from scRNA-seq analysis of samples collected as in Fig. 4a. Cells are coloured according to graph-based clusters (C1-C7)(*n* = 2255). Right panel: Bar charts showing the percentage of cells from different Seurat clusters in each sample. **b**, Venn diagrams showing the overlap between genes significantly downregulated (left) and upregulated (right) upon 4mM dm-αKG treatment in nESC (24 h) and aggregates (40 h). Shown are all genes differentially expressed genes with adj. p-val<0.05 from scRNA-seq experiments. Fisher’s exact test for both comparisons is p-val < 2.2e-16. **c**, Gene set enrichment analysis for differentially expressed genes between 40 h aggregate samples: 4 mM vs 0mM dm-αKG. Shown are most significantly enriched Hallmark terms. Normalised enrichment score is positive when terms are enriched in genes upregulated in 4 mM vs 0 mM. **d**, Left panel: representative IF images of Phospho-Histone H3-Ser10 (pH3S10) in 40 h aggregates treated with 0 or 4 mM of dm-αKG. Right panel: IF quantification showing the percentage of pH3Ser10+ve cells. Data is presented as the mean and data points of *N* = 2 biological replicates. Scale bar = 100 μm. **e**, Left panel: representative IF images of H3K27ac in 40 h aggregates treated with 0 or 4 mM of dm-αKG. Right panel: IF quantification showing H3K27ac intensity normalised to DAPI within the inner and outer layers of nuclei. *N* = 3 biological replicates, 40 h aggregates inner nuclei n = 863, 40 h aggregates outer nuclei n = 951, 40 h aggregates + 4mM dm-αKG inner nuclei n = 749, 40 h aggregates + 4mM dm-αKG outer nuclei n = 747. Box plot shows: center line: median; box minima and maxima: 25th and 75th percentiles; whiskers: 1.5x interquartile range, 75^th^ percentiles subtracted from 25^th^ percentiles. Scale bar = 100 μm. *P* values were calculated by two-sided Wilcoxon rank sum test. **f**, Expression of selected lineage-specific marker genes across samples related to Fig. 4f. The size of the circles represents the proportion of cells in the cluster for which expression of the gene is detected and colour encodes the scaled average expression. **g**, Distribution of pseudotime values in aggregate cells which are related to the in vivo epiblast (EPI). EPI pseudotime trajectories were identified from in vivo reference (Extended Data Fig. 4e). Source data

To assess transcriptional changes induced by dm-αKG, we performed scRNA-seq on 40 h aggregates. Cells originating from dm-αKG-treated aggregates established a unique transcriptional signature (Fig. 4e) and were particularly enriched within clusters C2, C4 and C6 (Extended Data Fig. 7a). Further analysis revealed a significant overlap between genes differentially expressed upon dm-αKG in aggregates and in the nES cell context (Extended Data Fig. 7b). Indeed, aggregates upregulated a number of metabolic regulators as well as apical polarity factors (Extended Data Fig. 7c). Together with imaging experiments, this suggests that dm-αKG induces metabolic rewiring and promotes efficient apical polarization. As in the nES cell context, these effects were not accompanied by increased proliferation (Extended Data Fig. 7d). We also found that dm-αKG treatment led to significant but mild H3K27ac depletion (Extended Data Fig. 7e). Despite these similarities with the nES cell context, in aggregates, increasing αKG resulted in only slight attenuation of naive pluripotency network, as exemplified by *DPPA5* and *DNTM3L* (Fig. 4f and Extended Data Fig. 7f). Indeed, upon integrating our scRNA-seq analysis with in vivo human reference datasets, we found that cells from dm-αKG-treated aggregates keep their Epi identity (Fig. 4g,h and Extended Data Fig. 7g). However, they retain a stronger pre-implantation signature than controls. In conclusion, in aggregates, dm-αKG-induced metabolic rewiring not only aids in apical polarization but also prevents cells from entering a post-implantation transcriptional signature. Moreover, within the 3D context, we observe not only metabolism being linked to epigenetics but also its coupling to cellular mechanics, in a process somewhat reminiscent of the in vivo morula compaction.

### dm-αKG facilitates blastoid induction and TE maturation

**Fig. 5:**
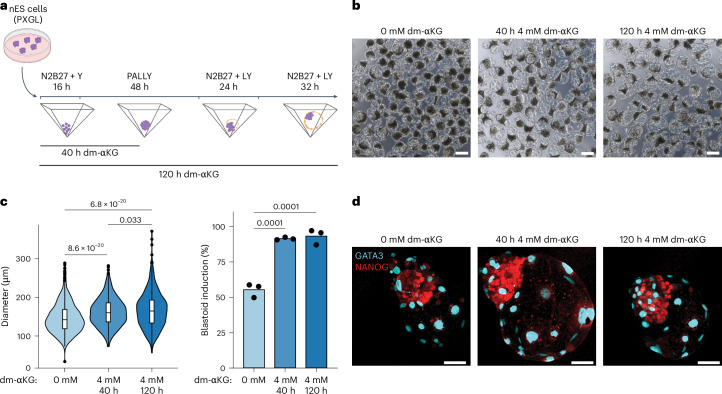
dm-αKG facilitates blastoid induction. **a**, A schematic of the blastoid induction protocol with 40 h and 120 h dm-αKG treatment. **b**, Representative brightfield images of day 5 blastoids untreated or treated for 40 h or 120 h with 4 mM dm-αKG. Scale bars, 200 μm. **c**, Quantification of blastoid diameter (left) and induction efficiency according to cavitation (right). Data are based on brightfield images of day 5 blastoids untreated or treated for 40 h or 120 h with 4 mM dm-αKG. Left: data are presented as violin plots with the median and 25th and 75 percentiles (±min–max) of *N* = 3 biological replicates, *n* = 737 (0 mM), 686 (4 mM, 40 h), 673 (4 mM, 120 h) blastoids. Right: data are presented as the mean and data points of *N* = 3 biological replicates. **d**, Representative IF images of 120 h blastoids with or without dm-αKG treatment (40 h or 120 h). Blastoids were stained for GATA3 (cyan) and NANOG (red). Shown is the maximum projection. Scale bars, 50 μm. *N* = 3 biological replicates. *P* values were calculated by one-way ANOVA tests with Bonferroni correction (**c**). Created with BioRender.com (**a**). Source data

**Fig. 6:**
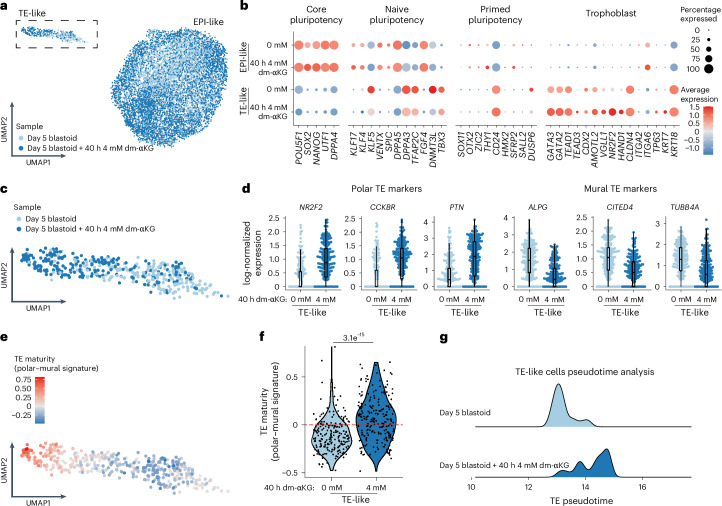
dm-αKG facilitates TE maturation during blastoid induction. **a**, UMAP plots from scRNA-seq analysis of samples treated for 40 h with 0 mM or 4 mM dm-αKG as in Fig. 5a. Cells are coloured according to sample (2 experiments, minimum 500 blastoids, *n* = 10,392 cells). The dotted box highlights TE-like cells. **b**, The expression of selected lineage-specific marker genes across samples further separated on the basis of Epi and TE signature. The size of the dots represents the proportion of cells in the indicated group expressing the given gene, and the colour encodes the scaled average expression. **c**, Magnification of UMAP (from **a**) focusing on TE-like cells. **d**, Combined beeswarm and box plots of log-normalized gene expression values of mature polar TE markers (pTE: *NR2F2*, *CCKBR* and *PTN*) and mural TE markers (mTE: *ALPG*, *CITED4* and *TUBB4A*). The sample type is shown with single cells plotted as dots; boxes span the interquartile range (IQR), the centre line indicates the median, and the whiskers extend to values within 1.5 times the IQR. **e**, UMAP as in **c** where the colour code depicts the enrichment of 25 pTE over 25 mTE marker signature. **f**, A violin plot showing the pTE–mTE signature as in **e** but separated by sample. The black dots are individual cells from 2 experiments and a minimum of 500 blastoids. **g**, The distribution of pseudotime values in blastoid cells, which are related to the in vivo TE. TE pseudotime trajectories were identified from in vivo reference (Extended Data Fig. 4e). *P* values were calculated by two-sided Wilcoxon rank-sum test (**f**). Source data

**Extended Data Fig. 8:**
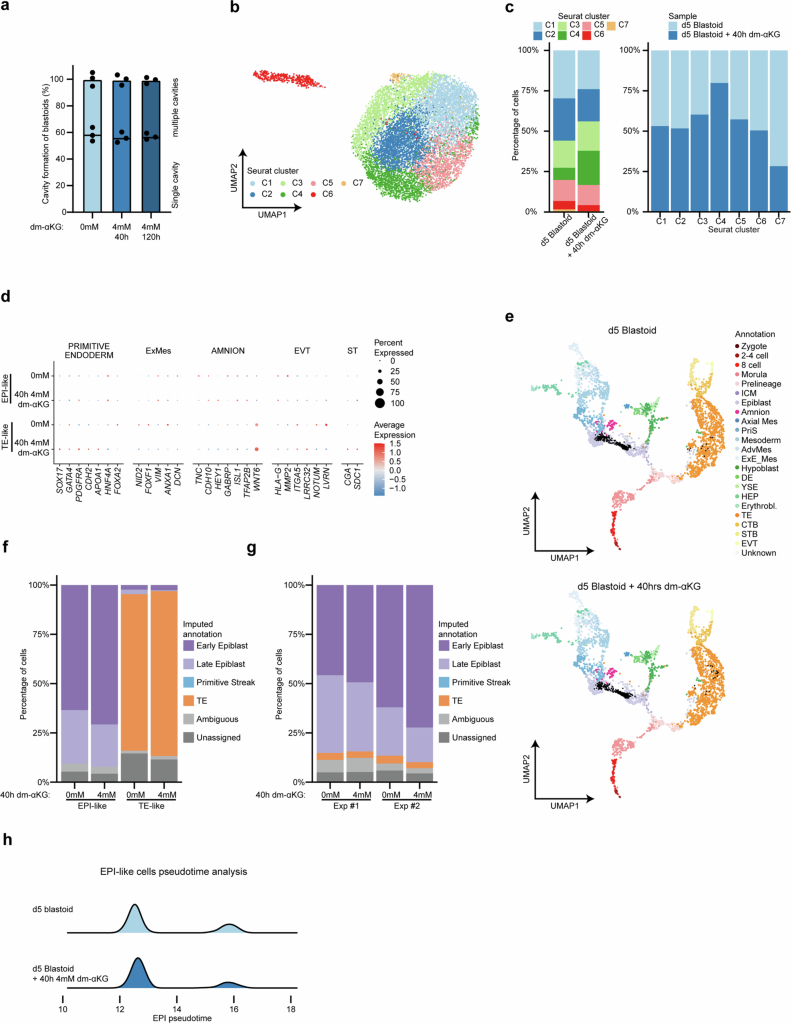
40h dm-αKG treatment of aggregates leads to improved blastoid development. **a**, Quantification of cavitated blastoid structures with singly cavity or multiple cavities. Data shows the mean percentages and data points of *N* = 3 biological replicates. **b**, UMAP plot from scRNA-seq analysis of samples collected as in Fig. 5a. Cells are coloured according to graph-based clusters (C1-C7)(*n* = 10392). **c**, Bar charts showing the percentage of cells from different Seurat clusters in each sample (left); and how samples contribute to Seurat clusters (right). **d**, Expression of selected lineage-specific marker genes across samples related to Fig. 6b. The size of the circles represents the proportion of cells in the indicated class for which expression of the gene is detected and colour encodes the scaled average expression. **e**, Integration of in vivo reference data (Fig. 2f) with in vitro d5 blastoids generated with 40 h 0 mM or 4mM dm-αKG treatment. Black dots show neighbourhoods of in vitro generated cells projected onto a reference UMAP. **f-g**, Imputed annotation of in vitro samples from reference in vivo dataset. Unassigned and ambiguous labels refer to cells with either none or with more than two imputed annotations respectively. Shown are cells from individual samples separated based on their EPI/TE-like marker gene expression as in Fig. 6a (**f**) or biological replicate (**g**). **h**, Distribution of pseudotime values in blastoid cells which are related to the in vivo epiblast (EPI). EPI pseudotime trajectories were identified from in vivo reference (Extended Data Fig. 4e). Source data

Together our data confirm that metabolic treatment aids in increasing the robustness of the first stage in TE induction. In line with this finding, blastoids obtained from such polarized aggregates were significantly larger (Fig. 5a–c and Extended Data Fig. 8a). Interestingly, we observed only a slight further increase in the size of blastoids when dm-αKG was kept throughout the blastoid induction protocol. This indicates that dm-αKG exerts most of its function at the early aggregate stage, despite the size difference becoming apparent only at day 5 of blastoid induction. IF on day 5 blastoids revealed that both control and dm-αKG treatment allows robust induction of GATA3-positive TE-like cells but also the maintenance of high levels of NANOG expression within the inner cells (Fig. 5d), which was not observed in 2D hiTS cell induction (Fig. 1d). This further highlights the importance of 3D cellular context as well as maintaining a signalling milieu compatible with Epi self-renewal. To validate our findings further, we performed scRNA-seq on day 5 blastoids induced from the 40 h 4 mM dm-αKG treatment. This revealed two clearly separated populations of cells on UMAP (Fig. 6a) where cells from control and dm-αKG treatment samples were largely intermingled (Extended Data Fig. 8b,c). Based on marker gene expression, we identified the larger population as Epi-like cells while the smaller cluster was assigned as the TE-like cells (Fig. 6b and Extended Data Fig. 8d). We further validated this annotation by integrating our data with the in vivo reference (Extended Data Fig. 8e,f). It is noteworthy that in our single-cell dissociation protocol there is a disproportional loss of fragile TE cells; thus, only relative lineage quantification could be performed. In line with IF experiments, we did not observe any significant difference in cell type ratios (Extended Data Fig. 8g). Focusing on marker gene expression, we found that Epi-like cells express similar levels of pluripotency factors, and in fact, only three genes were differentially expressed (adjusted *P* value <0.05 and |log_2_FC| >0.2) (Fig. 6b). Together this indicates that both control and dm-αKG treatment results in stable maintenance of the Epi lineage. Meanwhile, we observed that control and dm-αKG TE-like cells were unevenly distributed (Fig. 6c). In line with this, multiple markers related to TE maturity were differentially expressed. Indeed dm-αKG-treated TE-like cells upregulated markers of mature polar TE (pTE: *NR2F2*, *CCKBR* and *PTN*), while downregulating mural TE markers (mTE: *ALPG*, *CITED4* and *TUBB4A*) (Fig. 6d). To further validate this finding, we curated a list of 25 markers of both mature pTE and that of mTE based on published in vivo scRNA-seq analysis^10^. This, together with pseudotime trajectory analysis, confirmed that dm-αKG treatment results in blastoids with more cells expressing a mature TE transcriptional programme (Fig. 6e–g and Extended Data Fig. 8h). All in all, dm-αKG regulates the TE programme in blastoids, as it does in the 2D context. However, rather than attenuating pluripotency, it aids in initial polarization, aggregate patterning and TE maturation. One reason for these context-dependent effects might be that, unlike during the 2D experiments, we could not pretreat nES cells before aggregation because dm-αKG is not well tolerated by feeder cells.

**Extended Data Fig. 9:**
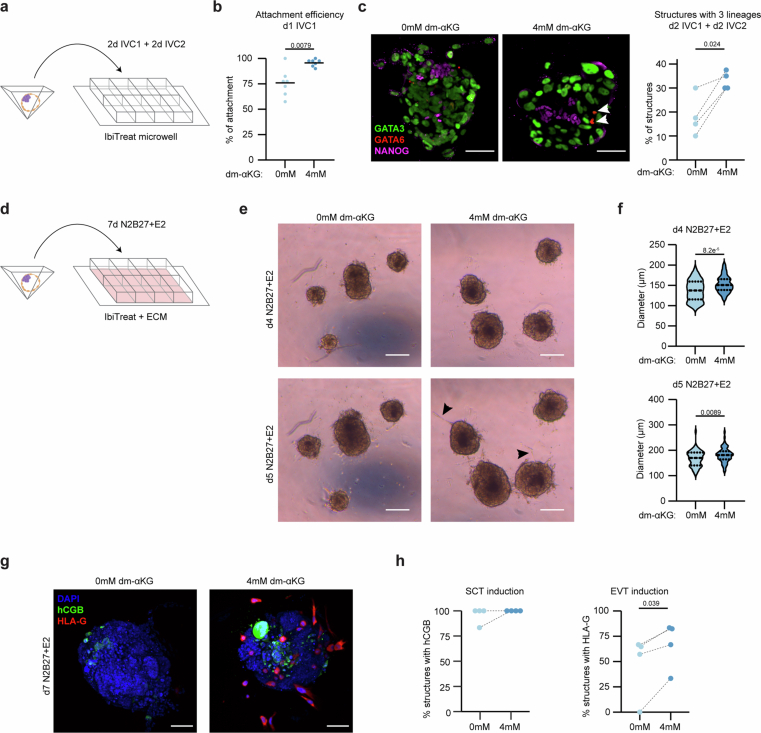
40h dm-αKG treatment of blastoids leads to improved attachment and trophoblast lineage differentiation. **a**, Schematic of blastoid attachment assays in IVC1-2 medium on IbiTreat µ-plates. **b**, Quantification of attachment efficiency 24 h after plating blastoids, treated with 40 h 0 mM or 4mM dm-αKG. Data shows the mean attachment percentages and data points of *N* = 7 biological replicates, n = 560. **c**, Left panel: representative IF images of NANOG, GATA3 and GATA6 in blastoids treated for 40 h with 0 or 4 mM of dm-αKG at d4 of attachment. White arrows indicate GATA6 single positive cells. Scale bar = 100 μm. Right panel: IF quantification. Data shows the mean percentages of structures with all 3 lineages (NANOG, GATA3 and GATA6) and data points of *N* = 4 biological replicates, n = 181. **d**, Schematic of blastoid attachment assays in N2B27 + E2 medium on IbiTreat µ-plates coated with ECM. **e**, Representative brightfield images of blastoids with or without 40 h 4mM dm-αKG treatment at d4 and d5 post attachment. Black arrows indicate extravillous trophoblast-like cells. Scale bar = 160 μm. **f**, Quantification of size (diameter) of blastoids treated for 40 h with 0 or 4 mM of dm-αKG at d4 and d5 of attachment. *N* = 4, n = 142 structures. Violin plot shows: center line: median; bottom and upper line: 25th and 75th percentiles. **g**, Representative IF images of DAPI, hCGB and HLA-G in blastoids treated for 40 h with 0 or 4 mM of dm-αKG at d7 of attachment. Scale bar = 100 μm. **h**, IF quantification of structures with syncytiotrophoblast- (SCT) and extravillous trophoblast- (EVT) like cells. Data shows the mean percentages of structures with hCGB or HLA-G positive cells and data points of *N* = 4 biological replicates, n = 115. *P* values were calculated by two-sided paired t-tests (**c**,**h**) or two-sided unpaired t-tests (**b,f**). Source data

Increased transcriptional TE-maturity of the blastoids with 40 h dm-αKG treatment suggests a more robust functionality of the TE lineage. To test this, we first assessed the capability of blastoids to attach to ibiTreat microwells (Extended Data Fig. 9a)^56^. On day 1 after plating, only 76.1% of control blastoids were attached, while this increased to 95.7% in the dm-αKG-treated group (Extended Data Fig. 9b). Further highlighting the improved functionality of blastoids, a significantly higher percentage of attached structures contained all three lineages (TE, GATA3; PrE, GATA6; Epi, NANOG) by day 4 of culture (Extended Data Fig. 9c). In vivo, pTE not only mediates embryo attachment but also undergoes further differentiation into specialized trophoblast lineages like the syncytiotrophoblast (SCT) and extravillous trophoblast (EVT). To assess the potential of blastoids to generate these two lineages, we adapted a previous protocol relying on the attachment of blastoids to a 3D extracellular matrix (ECM) (Extended Data Fig. 9d)^57^. Because this protocol does not allow one to monitor the blastoid attachment efficiency, we focused on measuring the size of the attached structures as well as their trophoblast lineage composition using IF. We found that blastoids treated with dm-αKG for 40 h formed significantly larger structures compared with control conditions, especially at day 4 of culture (Extended Data Fig. 9e,f). Importantly, by day 5, we observed the emergence of the first cells reminiscent of the EVT (Extended Data Fig. 9e). Next, we monitored the presence of hCGB-positive SCT- and HLA-G-positive EVT-like cells at day 7 of culture (Extended Data Fig. 9g). In both dm-αKG-treated and control conditions, nearly all structures contained some SCT-like cells (Extended Data Fig. 9h). There was high experimental variability in the efficiency to specify HLA-G-positive EVT-like cells in both control and treated blastoids (Extended Data Fig. 9h). Nevertheless, in line with increased TE functionality, dm-αKG treatment allowed significantly more structures to specify EVT-like cells (Extended Data Fig. 9h). Thus, consistent with the increased pTE signature in dm-αKG-treated blastoids, there is also improved functionality of the extra-embryonic lineages in extended in vitro cultures.

Collectively, these data support the hypothesis that metabolic rewiring during trophoblast induction functions as a positive feedback loop, stabilizing the cell fate decision as well as promoting TE maturity.

## Discussion

In this study, we demonstrate that there is a remarkable metabolic rewiring during trophoblast induction in vitro. In hiTS cells, we observe an accumulation of metabolites associated with glycolysis and the initial part of the TCA cycle, with αKG emerging as a key player. Transcriptomic analysis further confirmed that a similar process is likely to take place in the human blastocyst, where multiple TCA cycle enzymes are upregulated in the TE compared with the Epi. These findings are in line with increased oxygen consumption and mitochondrial content within the mouse TE compared with ICM^58^, as well as reported differences of the NAD(P)H autofluorescent signal between human TE and the ICM^59^. Presumably, this increased metabolic activity of the TE allows for energy-intensive processes such as blastocoel formation and expansion.

This metabolic rewiring is coupled to the cell fate decisions taking place at this time in development. Here, we demonstrate that increasing intracellular levels of αKG significantly enhances nES cell potential to differentiate into the TE lineage. Our study unveils an unexpected role for αKG, which facilitates hypoacetylation of the chromatin and attenuates the pluripotency network in human nES cells. Indeed, increased αKG levels, through feedback mechanisms common in metabolic circuits, result in reduced acetyl-CoA availability. Here, P300 is a putative sensor of the metabolic state of nES cells, allowing histone acetylation levels to respond to metabolic signals. The conservation of this regulatory axis across other mammalian species remains unclear and is in contrast to mouse naive ES cells, where elevated levels of αKG are instead associated with the stabilization of pluripotency, both transcriptionally and functionally^31,60^. Our data in human nES cells perhaps aligns more closely with observations in primed human ES (hES) cells, where dm-αKG enhances ectoderm induction^33^. However, unlike in primed hES cells, no histone methylation marks were significantly diminished upon dm-αKG treatment of nES cells at a global level. This includes H3K27me3, a known barrier for trophoblast specification in human^25,26^. One explanation for this discrepancy is that, in nES cells, steady-state levels of αKG are higher compared with primed hES cells. In such a context, histone demethylases may already be saturated with αKG. Importantly, we find that not only repressive histone methylation but also histone acetylation restricts the trophoblast fate in nES cells.

Our work also raises the question regarding the specificity in epi-metabolic regulation. While αKG affects global levels of acetylation, this results in a distinct phenotype with increased TE fate induction but reduced efficiency in nEnd specification or generation of off-target primitive streak and extra-embryonic mesoderm-like cells. The molecular basis for achieving such specificity remains largely unclear. Nevertheless, we propose that the dampened pluripotency network specifically potentiates trophoblast differentiation over other lineages. In line with this reasoning, the loss of OCT4 in mouse ES cells results in trophoblast marker expression, such as CDX2 (ref. ^4^). This observation is confirmed in the developing mouse embryo where OCT4 not only prevents trophoblast gene expression in the ICM but is also vital for PrE specification^61–63^. Therefore, it is likely that, in human nES cells, the naive pluripotency GRN restricts the expression of trophoblast-specific genes while simultaneously maintaining stability that is important for the initial stages of differentiation towards PrE.

dm-αKG treatment during blastoid formation proves effective in promoting apical polarization and TE maturation, thus highlighting its potential to enhance the robustness of this in vitro stem-cell-based embryo model. Notably, previous research has linked metabolism to mouse TE specification, particularly when glucose availability facilitates YAP activation^35^. The mode of action of dm-αKG is probably distinct from this axis of regulation as we do not observe overall nuclear YAP stabilization in nES cells or blastoids. Rather, our work highlights a surprising coupling between metabolism and cellular mechanics. Indeed, dm-αKG promotes apical polarization of aggregates in a process somewhat reminiscent of morula compaction in vivo. The mechanism underlying this observation remains unclear but may prove relevant for human development where blastomeres frequently fail to compact during human IVF embryo cultures^64^. Moreover, we find that early dm-αKG treatment results in enduring improvements to TE phenotypes. This is probably linked to either the enhanced early polarization or long-lasting epigenetic memory of early metabolite treatment.

Altogether, our work highlights that multiple parallel mechanisms couple cellular metabolism to other regulatory modalities affecting lineage specification and cellular competence. In early human development, the metabolic switch is predicted to coincide with blastocyst expansion. Our data in vitro substantiates the hypothesis that increased αKG levels in vivo form a positive feedback loop stabilizing the TE transcriptional programme. However, only challenging experiments on human embryos could shed light into this possibility. In summary, our findings underscore the critical role of metabolism in governing the competence of human naive pluripotent cells towards the TE lineage and highlight the context-dependent nature of such regulation.

## Methods

### Ethics statement

Our research complies with all relevant ethical regulations including the International Society for Stem Cell Research Guidelines for Stem Cell Research and Clinical Applications of Stem Cells. Experiments with human pluripotent stem cells and blastoids were approved by the Scientific Ethics Committee for Hovedstaden (H-21043866/94634). HNES1 line use is with agreement of the Steering Committee of the UK Stem Cell Bank (SCSC23-30). The WiCell line H9 (WA09) was used under the agreement 23-W0460. Extended blastoid culture was approved by the Scientific Ethics Committee for Hovedstaden (H-21043866/94634 and H-24048289). This work did not exceed a developmental stage normally associated with 14 consecutive days in culture after fertilization nor did it entail any implantation in vivo. Human embryos or gametes were not used in this study.

### Cell culture

Experiments were performed with the human embryonic stem cell lines H9 (WA09, WiCell) and HNES1 (Nichols Lab)^41^. Both cell lines were provided by the laboratory of J. Brickman. H9 cell line was chemically reset according to ref. ^12^. Cell lines were regularly tested for mycoplasma and were negative.

#### Human naive ES cell culture

Human nES cells were cultured on gelatin-coated plates including a feeder layer of gamma-irradiated DR4 mouse embryonic fibroblasts (ATCC, SCRC-1045) in tt2iLGö medium or PXGL medium. tt2iLGö medium consists of N2B27 basal medium supplemented with PD0325901 (1 μM, Axon MedChem, Axon 1408), CHIR99021 (0.3 μM, Axon MedChem, Axon 1386), Gö 6983 (2 μM, Axon MedChem, Axon 2466) and human leukaemia inhibitory factor (hLIF, 10 ng ml^−1^, Qkine, Qk036)^12^. PXGL medium consists of N2B27 basal medium supplemented with PD0325901 (1 μM, Axon MedChem, Axon 1408), XAV939 (2 μM, Merck Sigma-Aldrich, X3004), Gö 6983 (2 μM, Axon MedChem, Axon 2466) and hLIF (10 ng ml^−1^, Qkine, Qk036)^54^. N2B27 basal medium contains DMEM–F12 with GlutaMAX (50%, Thermo Fisher Scientific, 31331028), neurobasal medium (50%, Fisher Scientific, 11570556), N2 supplement (1×, Merck Sigma-Aldrich, SCM012), B27 supplement (0.5×, Thermo Fisher Scientific, 17504044), 2-mercaptoethanol (0.2 mM, Thermo Fisher Scientific, 31350010) and GlutaMAX (0.75 mM, Thermo Fisher Scientific, 25030024). Cells were cultured in hypoxic conditions (5% CO_2_, 5% O_2_) and passaged as single cells every 3–4 days. Cells were cultured for 24 h with Y27632 (10 μM, Axon MedChem, Axon 1683) after passaging. Medium was changed daily. Treatment of nES cells with dm-αKG, A-485 or UNC1999 was performed by directly diluting dimethyl 2-oxoglutarate (2 mM or 4 mM, Merck Sigma-Aldrich, 349631), A-485 (1 μM or 5 μM, Selleckchem, S8740) or UNC1999 (1 μM, BioNordika, CST-46080S) in the culture medium before medium change. Cell numbers were determined using the Via1-Cassette in the NucleoCounter NC-200 (Chemometec).

#### hiTS cell induction

hiTS cell induction was performed according to Dong et al., with minor modifications^20^. In brief, before the induction, nES cells were depleted from the feeder layer by incubating the single-cell suspension on 0.1% gelatin for 45–75 min. Human nES cells then were seeded at a density of 1.8 × 10^4^ cells cm^−2^ in a 6-well plate precoated with ECMatrix-511 Silk E8 Laminin Substrate (Merck Sigma-Aldrich, CC161) in tt2iLGö medium. After 24 h, the medium was changed to hiTS cell medium consisting of N2B27 supplemented with bovine serum albumin (BSA) (0.3%, Sigma, A3294), heat-inactivated foetal bovine serum (0.2%, Thermo Fisher Scientific, 12389782), insulin-transferrin-selenium-ethanolamine (ITS-X, 1×, Gibco, 51500), l-ascorbic acid (1.5 μg ml^−1^, Sigma, A8960-5G), recombinant human EGF (50 ng ml^−1^, Qkine, Qk011), CHIR99021 (2 μM, Axon MedChem, Axon 1386), A83-01 (0.5 μM, Axon MedChem, Axon 1421), SB431542 (1 μM, Axon MedChem, Axon 1661), valproic acid (0.8 mM, Axon MedChem, Axon 3127) and Y27632 (10 μM, Axon MedChem, Axon 1683). Cells were cultured in hypoxic conditions (5% CO_2_, 5% O_2_), and passaged as single cells or small clumps every 3–4 days. The medium was changed daily. Treatment of hiTS cell induction with dm-αKG, A-485 or UNC1999 was performed by directly diluting dimethyl 2-oxoglutarate (2 mM or 4 mM, Merck Sigma-Aldrich, 349631), A-485 (1 μM or 5 μM, Selleckchem, S8740) or UNC1999 (1 μM, BioNordika, CST-46080S) in the culture medium before medium change.

#### nEnd induction

nEnd induction was performed according to ref. ^16^ with minor modifications. In brief, before the induction, nES cells were depleted from the feeder layer by incubating the single-cell suspension on 0.1% gelatin for at least 45 min. Human nES cells then were seeded at a density of 2.2 × 10^4^ cells cm^−2^ in a 6-well plate precoated with ECMatrix-511 Silk E8 Laminin Substrate (Merck Sigma-Aldrich, CC161) in tt2iLGö medium. After 24 h, the medium was changed to RACL medium consisting of RPMI 1640 with GlutaMAX (Gibco, 61870044) with B27 minus insulin (Gibco, A1895601) supplemented with activin A (100 ng ml^−1^, Qkine, Qk005), CHIR99021 (3 μM) and hLIF (10 ng ml^−1^). Treatment of the nEnd induction with dm-αKG was performed by directly diluting dimethyl 2-oxoglutarate (2 mM or 4 mM, Merck Sigma-Aldrich, 349631) in the culture medium before medium change.

#### Blastoid formation

Blastoid experiments were performed according to Kagawa et al., with minor modifications^21^. In brief, nES cells cultured in PXGL were collected by incubation for 3 min with Accutase (Merck Sigma-Aldrich, A6964) and were depleted from the feeder layer by incubation on 0.1% gelatin for at least 60 min. Single cells were plated at a density of 80–85 cells per microwell of a 24-well AggreWell 400 plate (Stemcell Technologies, 34415) in N2B27 supplemented with BSA (0.3%, Sigma, A3294) and Y27632 (10 μM, Axon MedChem, Axon 1683) to aggregate for 16 h. Subsequently, half medium was changed with 2× PALLY consisting of N2B27 supplemented with BSA (0.3%, Sigma, A3294), PD0325901 (1 μM, Axon MedChem, Axon 1408), A83-01 (1 μM, Axon MedChem, Axon 1421), hLIF (10 ng ml^−1^, Qkine, Qk036), oleoyl-l-α-lysophosphatidic acid sodium salt (LPA, 1 μM, Merck Sigma-Aldrich, L7260) and Y27632 (10 μM, Axon MedChem, Axon 1683). Twenty-four hours later, half medium was changed with 1× PALLY. After 48 h of PALLY culture, blastoids were maintained for 56 h in LY medium consisting of N2B27 supplemented with BSA (0.3%, Sigma, A3294), LPA (1 μM, Merck Sigma-Aldrich, L7260) and Y27632 (10 μM, Axon MedChem, Axon 1683). Treatment of blastoid induction with dm-αKG was performed by directly diluting dimethyl 2-oxoglutarate (4 mM, Merck Sigma-Aldrich, 349631) in the culture medium. The medium was then equilibrated for 2–3 h at 37 °C in hypoxic conditions (5% CO_2_, 5% O_2_) before use.

#### Blastoid attachment assays

After 120 h blastoid induction, blastoids treated with 0 mM or 4 mM dm-αKG for 40 h were selected with a P10 pipette and transferred either on 4-well IbiTreat μ-plates (Ibidi, 80426) in IVC1 medium or on an ECM mix in 8-well IbiTreat μ-plates (Ibidi, 80826) in post-implantation medium. First, the IVC attachments were performed according to Shahbazi et al. with minor modifications^56^. In short, blastoids were cultured directly in IVC1 medium for 24 h before assessing attachment. After 48 h in IVC1, the medium was changed to IVC2 medium. IVC1 medium consists of advanced DMEM–F12 (Thermo Fisher Scientific, 11540446) supplemented with heat-inactivated foetal bovine serum (20%, Thermo Fisher Scientific, 12389782), GlutaMAX (2 mM, Thermo Fisher Scientific, 25030024), ITS-X (1×, Gibco, 51500), β-oestradiol (8 nM, Merck Sigma-Aldrich, E2758), progesterone (200 ng ml^−1^, Merck Sigma-Aldrich, P0130) and *N*-acetyl-l-cysteine (25 μM, Merck Sigma-Aldrich, A7250). IVC2 medium is composed of advanced DMEM–F12 (Thermo Fisher Scientific, 11540446) supplemented with knockout serum replacement (30%, Gibco, 10828028), GlutaMAX (2 mM, Thermo Fisher Scientific, 25030024), ITS-X (1×, Gibco, 51500), β-oestradiol (8 nM, Merck Sigma-Aldrich, E2758), progesterone (200 ng ml^−1^, Merck Sigma-Aldrich, P0130) and *N*-acetyl-l-cysteine (25 μM, Merck Sigma-Aldrich, A7250). Attachment efficiency was quantified for 7 biological replicates consisting of 80 structures each (*N* = 7, *n* = 560). Attached structures were collected 4 days after attachment.

The attachments on the ECM mix were performed according to Karvas et al. with guided modifications^57^. In brief, an 8-well IbiTreat μ-plate (Ibidi, 80826) was coated with an ECM mix consisting of Cultrex UltimaMatrix (80%, R&D System, BME001-05) and hES-cell-qualified Matrigel (20%, Corning, 11573560) for at least 1 h at 37 °C. Blastoids were transferred on the solidified ECM mix in post-implantation medium. Post-implantation medium or N2B27 + E2 consists of DMEM–F12 with GlutaMAX (50%, Thermo Fisher Scientific, 31331028), neurobasal medium (50%, Fisher Scientific, 11570556), N2 supplement (0.5×, Merck Sigma-Aldrich, SCM012), B27 supplement (0.5×, Thermo Fisher Scientific, 17504044), 2-mercaptoethanol (0.2 mM, Thermo Fisher Scientific, 31350010), GlutaMAX (2 mM, Thermo Fisher Scientific, 25030024), non-essential amino acids solution (1×, Gibco, 11140050), sodium pyruvate (1 mM, Gibco, 11360070) and β-oestradiol (10 nM, Merck Sigma-Aldrich, E2758). Medium was changed daily. At days 4 and 5, the size of the structures was measured (diameter, µm) (*N* = 4, *n* = 142). Structures were collected 7 days after attachment.

#### Real-time quantitative PCR

RNA was collected and extracted using the Qiagen RNeasy Mini Kit (Qiagen, 74104) with on-column DNAse treatment. Subsequently, cDNA was produced using Superscript III Reverse Transcriptase (Invitrogen, 18080093). RT-qPCR was performed using PowerUp SYBR Green Master Mix (Thermo Fisher Scientific, A25778) in a LightCycler480 (Roche). Quantification was performed by applying the comparative cycle threshold (Ct) method. Relative expression levels were normalized to *TBP*. The primers used during RT-qPCR analysis are summarized in Supplementary Table 5.

#### mRNA-seq

RNA samples were prepared as for RT-qPCR. Samples collected included nES cells treated with dimethyl sulfoxide (control, *n* = 4) or 4 mM dm-αKG (*n* = 2) or 1 μM A-485 (*n* = 2) for 24 h, and day 3 hiTS cells derived from these cells collected at day 3 of induction. Libraries for sequencing were prepared using 500 ng of total RNA and the NEBNext Poly(A) mRNA and NEBNext UltraII stranded RNA-seq library preparation kit. Ten cycles of amplification were performed. Samples were sequenced using NextSeq2000 P2 Reagents (100 cycles) v3 (Illumina) with paired-end 61-bp sequencing.

Raw sequencing data were demultiplexed and converted into FASTQ files using Illumina’s bcl2fastq (v2.20.0.422; RRID: SCR_015058), assigning an average of 31 million read pairs per library. Quality check was done with FastQC (v0.11.9; RRID: SCR_014583). Paired-end reads were aligned with STAR (v 2.7.2d; RRID: SCR_004463 (ref. ^65^)) to the reference genome GRCh38.p13 with gene annotations from GENCODE release 32 (RRID: SCR_014966 (ref. ^66^)) and option ‘--quantMode GeneCounts’ was set to count the number of reads per gene while mapping.

Differential gene expression analysis was performed using DESeq2 (1.36.0; RRID: SCR_015687 (ref. ^67^)) in R (v 4.2.1). Genes with fewer than ten raw read counts across samples were removed, and size factors were estimated for each sample. For nES cells and day 3 hiTS cells separately, DESeq was run with ‘design = ~treatment’, and results were extracted for dm-αKG versus control and P300i (A-485) versus control.

For GSEA, results for the P300i treatment were ranked by Wald statistics, and genes significantly up- or downregulated upon dm-αKG treatment (adjusted *P* value <0.05) were defined as separate gene sets. The R package fgsea (v 1.22.0; RRID: SCR_020938 (ref. ^68^)) was used to perform preranked GSEA with parameters ‘nPermSimple = 10000, eps = 0’, and defaults otherwise.

#### IF microscopy

Cells were cultured in Ibidi microwell plates and fixed with 2% paraformaldehyde for 10 min at room temperature. After fixation, paraformaldehyde solution was removed, and the samples were washed at least three times with 1× phosphate-buffered saline (PBS; Gibco, 14190144). The samples were then permeabilized for 20 min using 0.2% Triton X-100 (Merck Sigma-Aldrich, T8787) and afterwards blocked using blocking buffer containing 1% BSA (Merck Sigma-Aldrich, A7979), 0.1% Tween20 (Merck Sigma-Aldrich, P1379) and 10% normal donkey serum (Merck Sigma-Aldrich, D9663) in 1× PBS for at least 3 h. The samples were then incubated overnight at 4 °C with primary antibodies diluted in blocking buffer. The next day, samples were washed with 1× PBS containing 0.1% Tween20 (PBST) at least three times for 10 min each. After washing, the samples were incubated with secondary antibodies diluted in blocking buffer for 3 h in the dark at room temperature. The samples were then washed with PBST three times for 10 min each and afterwards mounted using Vectashield mounting medium (VWR, VECTH-1000) or 1× PBS. The antibodies used for IF assays are listed in Supplementary Table 5.

For blastoid and aggregate IF, the same protocol was followed with minor modifications. Namely, blastoid samples were fixed with 4% paraformaldehyde for 20 min at room temperature. Subsequently, the samples were washed for three times 10 min with PBST supplemented with BSA (0.3%). For image quantification of aggregates, sum projection of five consecutive confocal *z*-stacks was used. These stacks were 3 μm apart and at a central location of the aggregate. Nuclei were segmented in ilastik and curated in ImageJ using the DAPI signal. aPKCζ signal was used to identify the whole region of interest of each aggregate. For aPKCζ polarity quantification, the area between the end of the outer nuclei and the cell membrane was quantified and assigned as the apical signal. For each aggregate, the average apical intensity was divided by the remaining non-nuclear signal. For YAP and TAZ quantifications, signals were measured for each nucleus and categorized on the basis of their location either within the most outer layer or inner nuclei. Shown is the average IF intensity normalized to the outer cell signal.

After attachment, blastoid IF was performed following a similar protocol. In brief, the samples were fixed with 4% paraformaldehyde for 3 h at 4 °C, while gradually removing ECM. After fixation, paraformaldehyde solution was removed, and the samples were washed at least three times with 1× PBS. The samples were then permeabilized for 20 min using 0.3% Triton X-100 with 0.001% polyvinyl alcohol (PVA) (Merck Sigma-Aldrich, 363170) and afterwards blocked using blocking buffer containing 0.001% PVA, 1% BSA, 0.1% Tween20 and 10% normal donkey serum in 1× PBS for at least 5 h. The samples were then incubated for 48 h at 4 °C with primary antibodies diluted in blocking buffer. Subsequently, samples were washed with 1× PBS containing 0.1% Tween20 and 0.001% PVA (PBST) at least three times for 30 min each. After washing, the samples were incubated overnight with secondary antibodies diluted in blocking buffer in the dark at 4 °C. The samples were then washed with PBST three times for 30 min each and afterwards mounted using Vectashield mounting medium (VWR, VECTH-1000).

For H3K27ac/phospho-histone H3-Ser10 staining, IF was performed on aggregate cryosections. In this case, after fixation of the aggregates (see above), they were washed three times with PBST, transferred to 15% sucrose–PBS for 10 min at room temperature and then embedded in optimal cutting temperature compound and frozen on dry ice. Frozen blocks were stored at −80 °C and then sectioned at 8-µm thickness with a cryostat (Leica). Sections were placed on Superfrost Plus slides (ThermoFisher), air dried and stored at −80 °C.

For staining, slides were defrosted and air dried for 10 min. Sections were permeabilized for 15 min with 0.5% Triton X-100–PBST, rinsed three times with PBST and incubated for 1.5 h in blocking buffer. After blocking, primary antibodies in blocking buffer were added for 3.5 h, at room temperature. Next, samples were washed three times with PBST, followed by a 1-h incubation with secondary antibodies in blocking buffer. Finally, the sections were washed three times with PBST, which was supplemented with DAPI on the second wash (5-min incubation). Sections were mounted with SlowFade Diamond Antifade Mountant (Invitrogen) and a coverslip and subsequently sealed with nail varnish.

Image quantification of H3K27ac in cryosections was performed similar to YAP and TAZ in whole aggregates, with minor modifications. Here, the sum projection of three consecutive confocal *z*-stacks (*z*-size 2 µm) was used. After nuclear segmentation, outer versus inner cells of the aggregate were manually selected in each image and the fluorescence intensity of DAPI and H3K27ac was measured. Background signal was subtracted from the values, and H3K27ac intensity was normalized to DAPI. Shown is the average H3K27ac intensity in outer versus inner cells.

#### Gas chromatography–mass spectrometry time-of-flight (GC–MS-TOF) analysis

Cell samples were washed three times with 1× PBS before adding 1.0 ml ice-cold 90% methanol with labelled internal standards (Supplementary Table 5). The samples were collected by scraping the surface of the wells with a cell scraper and stored in −80 °C. Samples were thawed on ice with subsequent snap-freezing in liquid nitrogen, thawing on ice and vortexing for 10 s, which was repeated three times. Next, the samples were incubated on ice for 1 h, to facilitate the precipitation of proteins. Sample tubes were centrifuged at 16,000*g* or high speed for 15 min at 4 °C. Fifty microlitres of the resulting supernatant was transferred to gas chromatography vials and were dried at room temperature using a nitrogen evaporator. The dried metabolic extracts were stored at −20 °C until further analysis. The remaining pellet was used to measure protein concentration. Briefly, the pellets were resuspended in RIPA buffer (50 mM Tris–HCl pH 7.5, 150 mM NaCl, 1% NP-40, 0.1% SDS, 0.5% sodium deoxycholate and Sigma-Aldrich protease inhibitor Cocktail cOmplete). The lysates were incubated for 10 min on ice followed by sonication (4 °C, 8 cycles, each with 30-s pulses followed by 30 s rest in between) using a Bioruptor Plus. The lysates were centrifuged for 10 min at 20,000*g* at 4 °C. After centrifugation, the supernatants were used to measure protein content with the DC protein assay reagent (Bio-Rad, 5000111).

GC–MS-TOF analysis was performed with a Leco Pegasus BT GC/TOFMS instrument (Leco). Before the sample analysis, an automatic two-step derivatization reaction using a Gerstel MSP multisampler. In the first step, 12.5 µl of methoxamine reagent (2% in pyridine) was added to the dried extracts, and the mixture was incubated at 45 °C for 60 min. In the second step of the reaction, 12.5 µl of *N*,*O*-bis(trimethylsilyl)trifluoroacetamide with 1% trimethylchlorosilane was added to the same mixture, which was subsequently incubated under the same reaction conditions as before. Finally, to control for injection precision, 50 µl of hexane containing 10 mg l^−1^ of 4,4′-dibromooctafluorobiphenyl was added to the samples. The resulting silylated metabolites were separated on a Restek Rxi 5-ms column (pn: 13423-6850) with a helium flow of 1.2 ml min^−1^ and an inlet temperature of 270 °C. The temperature gradient started at 40 °C, where it was kept steady for 1 min. Next, temperature was increased at a pace of 20 °C min^−1^ until reaching 340 °C, where it was maintained for 3 min. Ions were generated by a −70 V electron beam at an ionization current of 2.0 mA, and 10 spectra s^−1^ were recorded in the mass range 50–750 *m*/*z*.

For data processing, raw files were converted into centroid mode and subsequently exported as netCDF files. The in-house Swedish Metabolome Centre (www.swedishmetabolomicscentre.se) GC–MS software was used for data extraction following a targeted approach. An in-house library, based on mass spectra and retention times of specific compounds, was used to obtain a target list of metabolites and their respective peak areas. The peak areas of target compounds were normalized to internal standards. For metabolite quantification, normalized peak areas were compared with a dilution series of standards. In addition, the normalized to internal compounds peak areas were further normalized to protein concentrations.

Methoxamine reagent and *N*,*O*-bis(trimethylsilyl)trifluoroacetamide with 1% trimethylchlorosilane were purchased from Thermo Scientific. Hexane, l-valine-d_8_, l-glutamic acid-^13^C_5_^15^N, succinic acid-2,2,3,3-d_4_ and 4,4′-dibromooctafluorobiphenyl were obtained from Sigma-Aldrich. Malic acid-^13^C_4_, citric acid-1,5,6-carboxyl-^13^C_3_, dl-2-hydroxyglutaric acid-^13^C_5_ and α-ketoglutaric acid-d_6_ were from Cambridge Isotope Laboratories. Standards aimed for quantification (d-glucose, d-fructose-6-phosphate, d-glucose-6-phosphate, l-lactic acid, pyruvic acid, citric acid, α-ketoglutaric acid, dl-α-hydroxyglutaric acid, succinic acid, dl-malic acid, fumaric acid, l-glutamine, *cis*-4-hydroxy-d-proline, d-ornithine, urea, l-tryptophan, l-alanine, l-glutamic acid, l-aspartic acid, l-proline, l-lysine, l-methionine, l-phenylalanine, l-serine, l-threonine, l-tyrosine and l-valine) were purchased from Sigma-Aldrich.

#### Liquid chromatography–quadrupole-time-of-flight mass spectrometry (LC–QTOF-MS) analysis of Acetyl-CoA

Cell samples were washed three times with 1× PBS before adding 0.5 ml ice-cold 90% methanol containing 0.5 µM acetyl-CoA-^13^C_2_ as internal standard. Then, 200 μl of the resulting supernatant was transferred to liquid chromatography vials and dried at room temperature using a nitrogen evaporator. The dried metabolic extracts were stored at −80 °C until further analysis.

LC–QTOF-MS analysis was performed with an Agilent 1290 Infinity II liquid chromatograph (Agilent) connected to a Bruker Tims TOF Pro-2 mass spectrometer (Bruker Daltonics). Metabolites were separated on an Atlantis Premier BEH-Z-HILIC 1.7 µm VanGuard Fit, 2.1 × 100 mm (Waters). The mobile phases were A: 10 mM NH_4_ acetate with 0.05 µM medronic acid), B: 90% acetonitrile with 10 mM NH_4_ acetate. The flow rate was 0.35 ml min^−1^. The gradient started with 90% of mobile phase B, and thereafter B was decreased to 60% in 5 min and further decreased to 30% in 2 min. After being held at 30% B, the column was reequilibrated back to 90% B. The column temperature was 40 °C, and acquisition was made with a vacuum-insulated probe-heated electrospray ionization source operated in positive ionization mode. The scan speed was 2 Hz, mass range 50–1,000 *m*/*z* and mass resolution about 50,000.

The peak area of acetyl-CoA was normalized to the internal standard. For metabolite quantification, normalized peak areas were compared with a dilution series of standards. In addition, the normalized to internal standard peak areas were further normalized to protein concentrations. Data processing was performed with Bruker Compass DataAnalysis version 6.1 software and TASQ 2023b (Bruker).

#### Histone posttranslational modification analysis

Samples were collected by 10 min of trypsinization. Subsequently, the samples were washed three times with 1 ml 1× PBS. After removing any remaining PBS, the samples were snap frozen. Samples were shipped to EpiQMAx and analysed as follows. Acid-extracted histones were processed according to a SP3 protocol as described previously^69^. However, proprietary steps developed by the EpiQMAx GmbH have been added to adjust the protocol for histone-specific aspects. Upon overnight digestion at 37 °C and 2,000*g* in a table-top thermomixer, samples were acidified by adding 5 µl of 5% trifluoroacetic acid (TFA) and quickly vortexed. Beads were immobilized on a magnetic rack, and peptides were recovered by transferring the supernatant to new PCR tubes. Samples were dried down using a vacuum concentrator and reconstituted by adding 12 µl 0.1% fluoroacetic acid (FA) to reach a peptide concentration of approximately 0.2 µg µl^−1^. mass spectrometry injection-ready samples were stored at −20 °C.

Approximately 200 ng of peptides from each sample were separated on a C18 column (bioZen 2.6 µm Peptide Polar-C18 150 ×0.075 mm Phenomenex) with a gradient from 5% B to 30% B (solvent A 0.1% FA in water, solvent B 80% acetonitrile (ACN), 0.1% FA in water) over 35 min at a flow rate of 300 nl min^−1^ (Vanquish Neo UHPLC-Systems, Thermo Fisher) and directly sprayed into a Exploris 240 mass spectrometer (Thermo Fisher Scientific). The mass spectrometer was operated in full-scan mode to identify and quantify specific fragment ions of N-terminal peptides of human histone 3.1 and histone 4 proteins. Survey full-scan mass spectrometry spectra (from *m*/*z* 250 to 1,600) were acquired with resolution 60,000 at *m*/*z* 400 (automatic gain control target of 3 × 10^6^). The mass spectrometric conditions were as follows: spray voltage, 1.8 kV; no sheath and auxiliary gas flow; heated capillary temperature, 300 °C.

Data analysis was performed with Skyline (version 20.2.0.343)^70^ by using doubly and triply charged peptide masses for extracted ion chromatograms. Peaks were selected manually. Heavy arginine-labelled SpikeTides (^13^C_6_; ^15^N_4_) were used to confirm the correct retention times and for signal normalization purposes, because all heavy standards were incorporated across all samples at the same concentration. Integrated peak values (total area MS1) were used for further calculations. Endogenous posttranslational modification (PTM) signals were normalized according to the variation of the signals of the spiked-in heavy standards. The percentage of each modification within the same peptide is derived from the ratio of this structural modified peptide to the sum of all isotopically similar peptides. Therefore, the total area MS1 value was used to calculate the relative abundance of an observed modified peptide as a percentage of the overall peptide. The unmodified peptide of histone 3.1 (amino acids 41–49) was used as indicator for total histone 3.1. Coeluting isobaric modifications were quantified using three unique MS2 fragment ions. Averaged integrals of these ions were used to calculate their respective contribution to the isobaric MS1 peak (for example, H3K36me3 and H3K27me2K36me1). To calculate the incorporation of precursor M2 [U-(13) C] from glucose into histone peptides, we first calculated the normal isotope distribution for a singly acetylated peptide in the control group. The incorporation of a heavy acetyl group shows a mass shift of 2 Da in the modified peptide. We took the intensity from precursor M [U-(12) C] and divided it by the intensity from precursor M2 [U-(13) C]. Further, the incorporation of precursor M2 [U-(13) C] in the treated samples was calculated by taking the precursor M2 [U-(13) C] intensity and subtracted by the ratio between precursor M [U-(12) C] from the treated group and divided by the normal isotope distribution ratio. This calculation was used to evaluate only the singly acetylated peptides. The relative percentage of the acetylated peptide generated by de novo acetylation was evaluated using the formula: (M2 [U-(13) C]/M2 [U-(13) C] + M [U-(12) C]) × 100.

#### ATAC-seq

ATAC-seq was performed as in ref. ^71^. In brief, nES cells were depleted from the feeder layer by incubating the single-cell suspension on 0.1% gelatin for 45–75 min. Cells then were seeded at a density of 1.8 × 10^3^ cells cm^−2^ in a 6-well plate precoated with ECMatrix-511 Silk E8 Laminin Substrate (Merck Sigma-Aldrich, CC161) in tt2iLGö medium supplemented with 0 or 4 mM dm-αKG. Twenty-four hours later, cells were trypsinized and resuspended in N2B27. After washing in PBS, 50,000 cells were lysed for 3 min on ice in lysis buffer (0.1% NP-40, 0.1% Tween20, 0.01% digitonin, 10 mM Tris–HCl pH 7.4, 10 mM NaCl and 3 mM MgCl). Lysis was stopped with ATAC-wash buffer (0.1% Tween20, 10 mM Tris–HCl pH 7.4, 10 mM NaCl and 3 mM MgCl). After pelleting the cells were resuspended in transposition mix (1× TD buffer (Illumina, cat. no. 20034197), 0.01% digitonin, 0.1% Tween20 in PBS, and TDE1 TD enzyme (Illumina, cat. no. 20034197)) and incubated for 30 min at 37 °C in a thermomixer set on 2,000*g*. Transposition was terminated with the DNA Binding Buffer DNA Clean and Concentrator-5 Kit (Zymo Research, cat. no. D4014), and DNA was purified. Libraries were prepared using barcoding adapters and the NebNext High-Fidelity 2x Master Mix (NEB, cat. no. M0541S). The cycling conditions used were as follows: 72 °C 5 min; 98 °C 30 s; 11× (98 °C 30 s, 63 °C 30 s); 72 °C 1 min. Libraries were purified twice using DNA Clean and Concentrator kit. Samples were sequenced using NextSeq2000 P2 Reagents (100 Cycles) v3 (Illumina) with paired-end 61-bp sequencing.

Raw sequencing data were demultiplexed and converted into FASTQ files using Illumina’s bcl2fastq (v2.20.0.422; RRID:SCR_015058), assigning an average of 38 million read pairs per library. These were further processed using the ENCODE ATAC-seq pipeline (v 2.2.2; https://github.com/ENCODE-DCC/atac-seq-pipeline; with ‘atac.genome_tsv’: https://storage.googleapis.com/encode-pipeline-genome-data/genome_tsv/v4/hg38.tsv, ‘atac.paired_end’: true, ‘atac.auto_detect_adapter’: true, and otherwise default parameters). One instance of the pipeline was run per treatment (control and dm-αKG treated), combining three replicates each. In brief, reads were trimmed and aligned to the reference genome with Bowtie 2 (ref. ^72^) and alignments filtered. Peak calling was performed by MACS2 (ref. ^73^) on each replicate, and reproducibility is assessed using the irreproducible discovery rate (IDR) framework.

Output from the two pipeline runs was merged as in the MoTrPAC ATAC-seq pipeline (https://github.com/MoTrPAC/motrpac-atac-seq-pipeline). In brief, optimal IDR peaks (idr.optimal_peak.narrowPeak.gz) from both runs were concatenated, peaks were truncated to 200 bp around summit, sorted and merged (that is, combining overlapping or ‘book-ended’ peaks using ‘bedtools merge’), and finally this ‘master peak file’ was intersected with tagalign files using bedtools coverage (bedtools v2.28.0; RRID:SCR_006646 (ref. ^74^)) to get counts for each replicate. Those peaks were further annotated using HOMER’s annotatePeaks.pl (v 4.11; with hg38 and default parameters^75^).

The count table was loaded into R (v 4.2.1), and differentially accessible peaks between dm-αKG treated and control were identified using DESeq2 (1.36.0) with default parameters. Peaks with an adjusted *P* value <0.01 and log_2_ fold change >1 were classified as more accessible in dm-αKG-treated, and likewise less accessible with adjusted *P* value <0.01 and log_2_ fold change <−1. Either peak set (with 793 and 2,479 peaks, respectively) was then checked for overrepresented motifs compared with the background set of all peaks using HOMER’s findMotifsGenome.pl (with parameters hg38 -size 200 -mask). Genomic Regions Enrichment of Annotations Tool (GREAT) analysis was performed using the R package (v 2.1.12^76^) for either peak set with background set of all peaks and parameters gene_sets = ‘msigdb:C2:CP’, tss_source = ‘TxDb.Hsapiens.UCSC.hg38.knownGene’.

#### scRNA-seq

Four 2D samples were collected by 10 min of trypsinization and dissociated into single cells. Single-cavity blastoids were collected by mouth pipetting. Then, 40-h aggregates and 120-h blastoids were dissociated into a single-cell suspension by incubation with a mixture of TrypLE 10× (1×, Thermo Fisher Scientific, A1217701) and Accutase (Merck Sigma Aldrich, A6964). Each sample was incubated with 0.5 μg of unique hashtag antibody for 20 min on ice. TotalSeq hashtag antibodies (A0251-A0256, BioLegend) were used to multiplex the samples^77^. All samples were sorted on a BD FACSymphony S6 (BD Bioscences) resulting in a total cell recovery of around 8,000 cells for the first experiment, 8,000 cells for the second experiment and 16,000 cells for the third experiment, which were then loaded onto a Chromium Next GEM chip (10x Genomics). Further steps of library preparation were performed according to the Chromium Next GEM Single Cell 3′ v3.1 user guide with the addition of the hashtag library for demultiplexing. Combined libraries were sequenced using the NextSeq2000 P2-100 kit (Illumina) with paired-end sequencing.

Initial processing of scRNA-seq data was performed using Cell Ranger (v 6.1.2; 10x Genomics). Dual-indexed RNA and single-indexed hashtag oligo (HTO) libraries were processed in separate instances of cellranger mkfastq, using Illumina’s bcl2fastq (v2.20.0.422). FASTQ files were aligned to the human reference genome (GRCh38, v 2020-A as provided by 10x Genomics) with cellranger count (--expect-cells = 8,000, 9,500 and 9,500), calling 8,316, 9,431 and 15,133 cells, respectively. Resulting filtered feature-barcode matrices were loaded into Seurat (v 4.3.0 (ref. ^78^)), excluding features that were detected in fewer than three cells. Next, RNA data were normalized with default LogNormalize method, and the HTO assay was normalized with centred log-ratio transformation. Seurat’s HTODemux function was used to assign single cells back to their sample origins, resulting in 7,001, 6,012 and 10,400 cells classified as singlets. Based on quality control plots, cells with more than 15% mitochondrial counts or less than 7,000 unique molecular identifiers (nCount_RNA; this threshold was set to 5,000 for the third experiment) were removed, retaining the following number of cells per sample: 1,158 nES cells, 900 nES cells + dm-αKG, 1,577 hiTS cells, 1,711 hiTS cells +dm-αKG (from sequencing run 1), 1,201 aggregates, 1,054 aggregates + dm-αKG, 426 blastoids, 1,092 blastoids + dm-αKG (from sequencing run 2), 3,900 blastoids, and 4,974 blastoids + dm-αKG (from sequencing run 3). These data from experiment 1 were subjected to standard Seurat processing using mostly default parameters unless indicated: FindVariableFeatures, ScaleData, RunPCA, RunUMAP (dims = 1:20), FindNeighbors (dims = 1:20) and FindClusters (resolution = 0.5), resulting in eight clusters. Aggregate samples (with and without dm-αKG) from experiment 2 were processed separately, blastoid samples were merged with those from experiment 3 (using merge(merge.data=TRUE)). To both, the aggregate subset and the merged blastoids, Seurat’s CellCycleScoring function was applied using the in-built lists of S and G2M phase markers (cc.genes.updated.2019). These were subsequently regressed out using ScaleData (vars.to.regress = c(‘S.Score’, ‘G2M.Score’), features = rownames(seur)), followed by RunPCA, RunUMAP (dims = 1:20) and FindNeighbors (dims = 1:20). FindClusters was run with a default resolution of 0.8 for the aggregates and with a resolution of 0.5 for the blastoids, both resulting in seven clusters. pTE and mTE were selected on the basis of ref. ^10^. For pTE markers, the top 25 genes of the *NR2F2* expression module were selected if they were upregulated in *NR2F2-*positive TE compared with *NR2F2-*negative (*NR2F2*, *SLC38A1*, *CCKBR*, *SP6*, *FYB1*, *VGLL1*, *CYP11A1*, *TINAGL1*, *TENM3*, *LGALS3*, *RHOBTB1*, *MRGPRX1*, *CBLB*, *CCR7*, *KCNN4*, *GREM2*, *MUC15*, *CYP19A1*, *S1PR2*, *PLEKHF1*, *PGF*, *LCMT1-AS2*, *MPP1*, *PWWP2B* and *GRAMD2B*). For mTE markers, the top 25 genes correlating in expression with *CDX2*/*S100A13*/*16*/*ATP6V0A4* were selected (*CDX2*, *RAB25*, *ATP6V0A4*, *FABP3*, *PRSS8*, *ATP6V1B1*, *GPRC5A*, *S100A13*, *LRP2*, *S100A6*, *ENPEP*, *FUOM*, *TAGLN2*, *GALNT10*, *SLC12A3*, *TMPRSS2*, *S100A16*, *TAX1BP3*, *PCYT2*, *LARGE2*, *ALPP*, *PTGES*, *CD53*, *DHCR24* and *PROM1*). On the subset of TE-like cells (as indicated in Fig. 5e), the expression of both mature pTE and mTE markers was scored using AddModuleScore; the difference of these is indicated as TE maturity (polar–mural signature).

To explore transcriptional differences in experiment 1 between Seurat clusters C1 (mainly nES cells) and C2 (mainly nES cells + dm-αKG), SeuratWrappers’ function RunPresto was called with parameters group.by = ‘seurat_clusters’, ident.1 = ‘C2’, ident.2 = ‘C1’, logfc.threshold = -Inf, min.pct = -Inf. This is a Presto-based implementation of FindMarkers that runs fast Wilcoxon rank-sum test and area under the receiver operating characteristics curve analysis. For preranked GSEA, results were filtered to genes that are expressed in more than 5% of cells in either C1 or C2 and ranked by AUC-0.5. Reactome gene sets were retrieved using msigdbr (v 7.5.1) with category = ‘C2’, subcategory = ‘CP:REACTOME’. fgsea (v 1.22.0) was run with parameters minSize = 15, maxSize = 500, gseaParam = 0 (that is, ‘classic’, not ‘weighted’ scoring scheme). For aggregates, markers were identified between treated and untreated cells using RunPresto with grouping by samples, and GSEA was performed in the same way, but on Hallmark gene sets, retrieved using msigdbr(category = ‘H’).

The human embryo reference was established utilized fastMNN from Batchelor (v.1.6.2)^79^, through the integration of previously published datasets, encompassing six human embryonic datasets spanning from early-stage in vitro cultured human blastocysts^9,10,22,46^ to 3D in vitro cultured human blastocysts^45^, and up to CS7 human gastrula^44^, as described in ref. ^43^. The pseudotime of the reference was calculated as described in ref. ^43^. In detail, the pseudotimes of the reference were inferred using Slingshot (v.2.6.0)^80^ based on the 2D UMAP embeddings of the reference. The raw cell aggregation, projection onto the human embryo reference, cell identity prediction and pseudotime inferring, were performed using the Early Embryogenesis Prediction tool^43^. In brief, raw cell counts were aggregated within neighbourhood nodes, computed using ‘makeNhoods’ with from miloR(v1.2.0)^81^, resulting in 471, 329 and 813 representative neighbourhoods for the different experiments. The aggregated counts matrix was then normalized, and corrected by mutual nearest neighbour (MNN) identification to remove batch effects, followed by transformation to the reference UMAP by using the umap_transform function from the R package uwot (v.0.1.14)^82^. Cell identities were predicted by an SVM classifier generated on 20 UMAP-transformed latent spaces, and the pseudotime of query cells was determined using *k*-nearest neighbour clustering (*k* = 5) with reference cells belonging to the same lineages calculated in the top 50 PCA-corrected latent spaces.

In addition, for experiment 1, the developmental stage of the representative neighbourhoods was inferred by a *k*-nearest neighbours algorithm (with *k* = 3, as implemented in the Rfast package (v 2.0.7)) based on the Euclidean distance in the 2D UMAP embedding. These labels were propagated to all cells contributing to the neighbourhood. As there is not a 1:1 mapping between cells and neighbourhoods, final labels were assigned as follows. If >50% of labels for one cell agree, this is used as the final label; if two labels are exactly 50% each, both are used (for example, ‘E6/E7’); all other cases are considered ‘ambiguous’; if a cell is not included in any neighbourhood, it is ‘unassigned’.

#### Microscopy

Brightfield images were acquired with a Leica DM IL LED microscope. IF images were made with the Deltavision Widefield screening microscope, Zeiss 880 airyscan, Leica Stellaris and Leica SP8 confocal microscope. All images were analysed using Fiji (ImageJ) and/or Ilastik^83^.

#### Statistics and reproducibility

scRNA-seq for the 120 h blastoid samples was performed in two independent experiments. scRNA-seq for hiTS and nES cells and 40 h aggregates were performed once. ATAC-seq, mRNA-seq, GC–MS-TOF and histone PTM quantification were each performed once, with three, two, three and three biological replicates, respectively. All other experiments in this study were repeated three times, unless stated otherwise in the figure legends. Statistics were performed using GraphPad Prism 10.4.1 (627) or R (v 4.2.1; RRID: SCR_001905). Normality and equal variances were tested where necessary. No statistical method was used to determine sample size. For the three scRNA-seq runs, 1,315, 3,419 and 4,733 droplets were excluded because they could not be assigned to one sample by Seurat’s HTODemux. In addition, 1,655, 2,239 and 1,526 cells were excluded during quality control for having more than 15% mitochondrial counts or less than 7,000, 7,000 or 5,000 unique molecular identifiers, respectively. No other data were excluded. The experiments were not randomized. The investigators were not blinded to allocation during the experiments and outcome assessment.

### Reporting summary

Further information on research design is available in the Nature Portfolio Reporting Summary linked to this article.

## Online content

Any methods, additional references, Nature Portfolio reporting summaries, source data, extended data, supplementary information, acknowledgements, peer review information; details of author contributions and competing interests; and statements of data and code availability are available at 10.1038/s41556-025-01658-1.

## Supplementary information

Reporting Summary

Supplementary Table 1 GSEA of nES cells with 0 or 4 mM dm-αKG.

Supplementary Table 2 Histone posttranslational modification quantifications of nES cells with 0 or 4 mM dm-αKG.

Supplementary Table 3 Differential gene expression analysis of nES cells with 0 or 4 mM dm-αKG.

Supplementary Table 4 ^13^C-glucose (U-^13^C-glucose) histone acetylation labelling data.

Supplementary Table 5 Methods for gas chromatography–mass spectrometry (internal controls), primer sequences and antibodies.

## Source data

Source Data Fig. 1 Statistical source data.

Source Data Fig. 2 Statistical source data.

Source Data Fig. 3 Statistical source data.

Source Data Fig. 4 Statistical source data.

Source Data Fig. 5 Statistical source data.

Source Data Fig. 6 Statistical source data.

Source Data Extended Data Fig. 1/Table 1 Statistical source data.

Source Data Extended Data Fig. 2/Table 2 Statistical source data.

Source Data Extended Data Fig. 3/Table 3 Statistical source data.

Source Data Extended Data Fig. 4/Table 4 Statistical source data.

Source Data Extended Data Fig. 5/Table 5 Statistical source data.

Source Data Extended Data Fig. 6/Table 6 Statistical source data.

Source Data Extended Data Fig. 7/Table 7 Statistical source data.

Source Data Extended Data Fig. 8/Table 8 Statistical source data.

Source Data Extended Data Fig. 9/Table 9 Statistical source data.

## Data Availability

Sequencing data that support the findings of this study have been deposited in the Gene Expression Omnibus (GEO) under accession code GSE247634. Datasets utilized in the in vivo reference include six human embryonic datasets covering various stages of embryogenesis: Yan et al. (GSE36552), Petropoulos et al. E-MTAB-(3929), Meistermann et al. (PRJEB30442), Yanagida et al. (GSE171820), Xiang et al. (GSE136447) and Tyser et al. E-MTAB-(9388). The processed dataset, including predicted annotations, UMAP and sorted cell counts, is available at https://petropoulos-lanner-labs.clintec.ki.se/dataset.download.html. Source data are provided with this paper. All other data supporting the findings of this study are available from the corresponding author on reasonable request.
