# Supplementary material for: α-Ketoglutarate promotes trophectoderm induction and maturation from naive human embryonic stem cells
Source: Nat Cell Biol. 2025 Apr 23;27(5):749–61. doi: 10.1038/s41556-025-01658-1 (PMC12081308; doi:10.1038/s41556-025-01658-1)
Supplement: Supplementary file 1 — Reporting Summary [file 41556_2025_1658_MOESM1_ESM.pdf]

Corresponding author(s): Jan Zylicz
Last updated by author(s): Feb 24, 2025

Reporting Summary

Nature Portfolio wishes to improve the reproducibility of the work that we publish. This form provides structure for consistency and transparency in reporting. For further information on Nature Portfolio policies, see our Editorial Policies and the Editorial Policy Checklist.

Statistics

For all statistical analyses, confirm that the following items are present in the figure legend, table legend, main text, or Methods section.

n/a	Confirmed	
☐	☒	The exact sample size (*n*) for each experimental group/condition, given as a discrete number and unit of measurement
☐	☒	A statement on whether measurements were taken from distinct samples or whether the same sample was measured repeatedly
☐	☒	The statistical test(s) used AND whether they are one- or two-sided<br>*Only common tests should be described solely by name; describe more complex techniques in the Methods section.*
☒	☐	A description of all covariates tested
☐	☒	A description of any assumptions or corrections, such as tests of normality and adjustment for multiple comparisons
☐	☒	A full description of the statistical parameters including central tendency (e.g. means) or other basic estimates (e.g. regression coefficient) AND variation (e.g. standard deviation) or associated estimates of uncertainty (e.g. confidence intervals)
☐	☒	For null hypothesis testing, the test statistic (e.g. *F*, *t*, *r*) with confidence intervals, effect sizes, degrees of freedom and *P* value noted<br>*Give P values as exact values whenever suitable.*
☒	☐	For Bayesian analysis, information on the choice of priors and Markov chain Monte Carlo settings
☒	☐	For hierarchical and complex designs, identification of the appropriate level for tests and full reporting of outcomes
☒	☐	Estimates of effect sizes (e.g. Cohen's *d*, Pearson's *r*), indicating how they were calculated

Our web collection on statistics for biologists contains articles on many of the points above.

Software and code

Policy information about availability of computer code

Data collection

No software was used for data collection.

Data analysis

Base calling and demutiplexing: bcl2fastq (v2.20.0.422; RRID:SCR_015058)
Read quality control: FastQC (v0.11.9; RRID:SCR_014583)

Alignment (RNAseq): STAR (v 2.7.2d; RRID:SCR_004463)

ENCODE ATAC-seq pipeline (v 2.2.2; https://github.com/ENCODE-DCC/atac-seq-pipeline)within that:
Bowtie2 (v 2.3.4.3; RRID:SCR_016368)
Picard (v 2.20.7; RRID:SCR_006525)
MACS2 (v 2.2.4; RRID:SCR_013291)
bedtools (v 2.29.0; RRID:SCR_006646)
idr (v 2.0.4.2)

combine peak files from different conditions and get counts: bedtools (v v2.28.0; RRID:SCR_006646)
annotate peaks and find enriched motifs: HOMER (v 4.11; RRID:SCR_010881)

Cell Ranger (v 6.1.2; RRID:SCR_017344)

R (v 4.2.1; RRID:SCR_001905)

R packages:
Differential gene expression; differential accessible peaks): DESeq2 (1.36.0; RRID:SCR_015687)
preranked gene set enrichment analysis: fgsea (v 1.22.0; RRID:SCR_020938)
rGREAT (v 2.1.12)
Seurat (v 4.3.0)
SeuratWrappers (v 0.3.1)
presto (v 1.0.0)
msigdbr (v 7.5.1)
batchelor (v.1.6.2)
miloR (v1.2.0)
uwot (v.0.1.14)
Rfast (v 2.0.7)

GraphPad Prism (v 10.1.0(316))
Fiji/Image J (v 2.9.0/1.53t)
ilastik (v 1.3.3 and 1.4.1)

Bruker Compass DataAnalysis version 6.1
TASQ 2023b (Bruker)
Skyline (version 20.2.0.343)

For manuscripts utilizing custom algorithms or software that are central to the research but not yet described in published literature, software must be made available to editors and reviewers. We strongly encourage code deposition in a community repository (e.g. GitHub). See the Nature Portfolio guidelines for submitting code & software for further information.

Data

Policy information about availability of data

All manuscripts must include a data availability statement. This statement should provide the following information, where applicable:

- Accession codes, unique identifiers, or web links for publicly available datasets
- A description of any restrictions on data availability
- For clinical datasets or third party data, please ensure that the statement adheres to our policy

All NGS datasets generated in this study are available on GEO GSE247634. Datasets utilized in the in vivo reference include 6 human embryonic datasets covering various stages of embryogenesis: Yan et al. (GSE36552), Petropoulos et al. (E-MTAB-3929), Meistermann et al. (PRJEB30442), Yanagida et al. (GSE171820), Xiang et al. (GSE136447), Tyser et al. (E-MTAB-9388). The processed dataset, including predicted annotations, UMAP and sorted cell counts, can be retrieved from https://petropoulos-lanner-labs.clintec.ki.se/dataset.download.html.

Research involving human participants, their data, or biological material

Policy information about studies with human participants or human data. See also policy information about sex, gender (identity/presentation), and sexual orientation and race, ethnicity and racism.

Reporting on sex and gender	N/A
Reporting on race, ethnicity, or other socially relevant groupings	N/A
Population characteristics	N/A
Recruitment	N/A
Ethics oversight	N/A

Note that full information on the approval of the study protocol must also be provided in the manuscript.

Field-specific reporting

Please select the one below that is the best fit for your research. If you are not sure, read the appropriate sections before making your selection.

☒ Life sciences ☐ Behavioural & social sciences ☐ Ecological, evolutionary & environmental sciences

For a reference copy of the document with all sections, see nature.com/documents/nr-reporting-summary-flat.pdf

Life sciences study design

All studies must disclose on these points even when the disclosure is negative.

Sample size	No statistical method was used to determine sample size. Sample sizes were chosen based on previous experience or similar studies (Development: https://doi.org/10.1242/dev.180620; Nature Cell Biology: https://doi.org/10.1038/s41556-022-00916-w).

All sample sizes are indicated in the figure legends or the manuscript.
For stem cells, RT-qPCR, immunostaining blastoids, experiments were performed in three independent replicates unless stated otherwise in the figure legends.
GC/MS-TOF analysis was performed for three biological replicates.
hPTM quantification was performed for three or four biological replicates.

Data exclusions	No data was excluded for analyses unless stated otherwise in the methods.
Replication	scRNA-seq was performed three times with no biological replicates except for the 120h blastoid samples for which 2 biological replicates were sequenced. ATAC-seq, mRNA-seq, GC/MS-TOF and hPTM quantification were performed once with at least 2 biological replicates. All other experiments in this study were repeated three times, unless stated otherwise in the figure legends.
Randomization	Allocation of cell culture samples to treated or control groups was randomized in cell culture wells. Control and treatment samples were collected and analyzed concurrently. Representative images and images for immunofluorescence analysis were acquired with no particular bias.
Blinding	Experimental design and sample collection was performed by the same individual. Analysis of scRNA-seq, ATAC-seq, GC/MS-TOF and hPTM quantification was performed by different investigators. No further blinding was required as the reported analysis do not involve procedures susceptible to investigator bias.

Reporting for specific materials, systems and methods

We require information from authors about some types of materials, experimental systems and methods used in many studies. Here, indicate whether each material, system or method listed is relevant to your study. If you are not sure if a list item applies to your research, read the appropriate section before selecting a response.

Materials & experimental systems

n/a	Involved in the study	
☐	☒	Antibodies
☐	☒	Eukaryotic cell lines
☒	☐	Palaeontology and archaeology
☒	☐	Animals and other organisms
☒	☐	Clinical data
☒	☐	Dual use research of concern
☒	☐	Plants

Methods

n/a	Involved in the study	
☒	☐	ChIP-seq
☒	☐	Flow cytometry
☒	☐	MRI-based neuroimaging

Antibodies

Antibodies used

Primary antibodies:
anti-GATA3 1 in 250 (2D); 1 in 500 (3D) Abcam, ab199428
anti-NANOG 1 in 250 (2D); 1 in 200 (3D) Abcam, ab173368
anti-BRACHYURY 1 in 250 R&D Systems, AF2085
anti-aPKCζ 1 in 100 Santa Cruz Biotechnology, sc17781
anti-YAP1 1 in 100 Proteintech, 13584-1-AP
anti-H3K27Ac 1 in 500 Active Motif, 39685
anti-GATA4 1 in 250 eBioscience, 14-9980-82
anti-GATA6 1 in 100 R&D Systems, AF1700
anti-P300 1 in 400 Cell Signaling Technologies, CST-86377T
anti-SOX2 1 in 200 R&D Systems, AF2018
anti-HLA-G 1 in 200 Santa Cruz Biotechnology, sc21799
anti-hCGbeta 1 in 200 Abcam, ab53087

Secondary antibodies:
Donkey anti-Rabbit, Alexa Fluor Plus 488, Highly Cross-Adsorbed 1 in 500 Fischer Scientific, A32790
Donkey anti-Mouse, Alexa Fluor® 568 Highly Cross-Adsorbed 1 in 500 Fischer Scientific, 10236683
Donkey anti-Goat, Alexa Fluor™ 568, Cross-Adsorbed 1 in 500 Fischer Scientific, A11057
Donkey anti-Rabbit, Alexa Fluor® Plus 647 Highly Cross-Adsorbed 1 in 250 Fischer Scientific, 16239260
Donkey anti-Rat, Alexa Fluor® 488 Highly Cross-Adsorbed 1 in 500 Fischer Scientific, A21208
Donkey anti-Goat, Alexa Fluor™ 647, Cross-Adsorbed 1 in 250 Fischer Scientific, A21447
Donkey anti-Mouse, Alexa Fluor® 647 Highly Cross-Adsorbed 1 in 250 Fischer Scientific, A31571

Hashtag antibodies:
TotalSeq™-A0251 anti-human Hashtag 1 Antibody, mouse, Biolegend 394601
TotalSeq™-A0252 anti-human Hashtag 1 Antibody, mouse, Biolegend 394603
TotalSeq™-A0253 anti-human Hashtag 1 Antibody, mouse, Biolegend 394605
TotalSeq™-A0254 anti-human Hashtag 1 Antibody, mouse, Biolegend 394607
TotalSeq™-A0255 anti-human Hashtag 1 Antibody, mouse, Biolegend 394609
TotalSeq™-A0256 anti-human Hashtag 1 Antibody, mouse, Biolegend 394611

Validation

All antibodies were previously validated either by vendors or publications.
GATA3 Abcam, ab199428; cited in 27 publications
https://www.abcam.com/en-us/products/primary-antibodies/gata3-antibody-epr16651-chip-grade-ab199428?srsltid=AfmBOoqUQitC-WHY5jPBntdTF1A8LBNF7DeeS8RwNnBRg-vsD6oKLADj
NANOG Abcam, ab173368; cited in 7 publications
https://www.abcam.com/en-us/products/primary-antibodies/nanog-antibody-23d2-3c6-ab173368?srsltid=AfmBOorXd066aJQVKmo__GQuvXYWn9f59nbZD4sjXpDL6bpsyZzs_TwE

BRACHYURY R&D Systems, AF2085; cited in 269 publications
https://www.rndsystems.com/products/human-mouse-brachyury-antibody_af2085

aPKCζ Santa Cruz Biotechnology, sc17781; cited in 142 publications
https://www.scbt.com/p/pkc-zeta-antibody-h-1?srsltid=AfmBOooV_P6tE-18E2CjL_rHO_EotaXcl8FipsZVHCZKyclJo8ev8ErT

YAP1 Proteintech, 13584-1-AP; cited in 301 publications
https://www.ptglab.com/products/YAP1-Antibody-13584-1-AP.htm?srsltid=AfmBOorWDTLRKpBQeu9UwfQPC3bvLos2E45otHPKTvHSgKoTn_rAmRCC

H3K27Ac Active Motif, 39685 clone: MABI 0309; cited in 18 publications
https://www.activemotif.com/catalog/details/39685

GATA4 (eBioEvan), eBioscience, 14-9980-82; cited in 14 publications
https://www.thermofisher.com/antibody/product/Gata-4-Antibody-clone-eBioEvan-Monoclonal/14-9980-82

GATA6 R&D Systems, AF1700; cited in 139 publications
https://www.rndsystems.com/products/human-gata-6-antibody_af1700

P300 Cell Signaling Technologies, CST-86377T; cited in 5 publications
https://www.cellsignal.com/products/primary-antibodies/p300-d8z4e-rabbit-mab/86377?srsltid=AfmBOoqvWUL6aBLcoeVpT5lTarFvSw7r2n99oZMJmm-5Cud9OeMrJwQd

SOX2 R&D Systems, AF2018; cited in 379 publications
https://www.rndsystems.com/products/human-mouse-rat-sox2-antibody_af2018

HLA-G Santa Cruz Biotechnology, sc21799; cited in 83 publications
https://www.scbt.com/p/hla-g-antibody-4h84?srsltid=AfmBOop7O1txDcs4k5qpLDl7qRcXDg-yP_eTEqRY5GAfbzCnXyNDpfYE

hCGbeta Abcam, ab53087; cited in 13 publications
https://www.abcam.com/en-us/products/primary-antibodies/hcg-beta-antibody-ab53087?srsltid=AfmBOorGNLuEshAe4yYuqhKlpfeBvB8bpnjuo3YULQR6hdaYiFfayp-v

TotalSeq™-A0251 anti-human Hashtag 1 Antibody, mouse, Biolegend 394601; cited in 16 publications
https://www.biolegend.com/en-us/products/totalseq-a0251-anti-human-hashtag-1-16080
TotalSeq™-A0252 anti-human Hashtag 1 Antibody, mouse, Biolegend 394603; cited in 17 publications
https://www.biolegend.com/en-us/products/totalseq-a0252-anti-human-hashtag-2-antibody-16081
TotalSeq™-A0253 anti-human Hashtag 1 Antibody, mouse, Biolegend 394605; cited in 14 publications
https://www.biolegend.com/en-us/products/totalseq-a0253-anti-human-hashtag-3-antibody-16084
TotalSeq™-A0254 anti-human Hashtag 1 Antibody, mouse, Biolegend 394607; cited in 15 publications
https://www.biolegend.com/en-us/products/totalseq-a0254-anti-human-hashtag-4-antibody-16086
TotalSeq™-A0255 anti-human Hashtag 1 Antibody, mouse, Biolegend 394609; cited in 12 publications
https://www.biolegend.com/en-us/products/totalseq-a0255-anti-human-hashtag-5-antibody-16088
TotalSeq™-A0256 anti-human Hashtag 1 Antibody, mouse, Biolegend 394611; cited in 10 publications
https://www.biolegend.com/en-us/products/totalseq-a0256-anti-human-hashtag-6-antibody-16089

Eukaryotic cell lines

Policy information about cell lines and Sex and Gender in Research

Cell line source(s)

H9 (WA09, WiCell) and HNES1 (Nichols Lab, DOI: 10.1016/j.stemcr.2016.02.005, PMID: 26947977) both provided by the Brickman lab. Mouse embryonic fibroblasts (ACTT, SCRC-1045).

Authentication

Cell lines were not further authenticated.

Mycoplasma contamination

All the cell lines are tested negative for mycoplasma contamination.

Commonly misidentified lines
(See ICLAC register)

No commonly misidentified lines were used in this study.

Plants

Seed stocks	N/A
Novel plant genotypes	N/A
Authentication	N/A
